# Autoimmune Dysregulation and Purine Metabolism in Adenosine Deaminase Deficiency

**DOI:** 10.3389/fimmu.2012.00265

**Published:** 2012-08-27

**Authors:** Aisha Vanessa Sauer, Immacolata Brigida, Nicola Carriglio, Alessandro Aiuti

**Affiliations:** ^1^San Raffaele Telethon Institute for Gene TherapyMilan, Italy; ^2^Università degli Studi di Roma Tor VergataRome, Italy

**Keywords:** adenosine deaminase, severe combined immunodeficiency, ADA-SCID, autoimmunity, gene therapy

## Abstract

Genetic defects in the adenosine deaminase (*ADA*) gene are among the most common causes for severe combined immunodeficiency (SCID). ADA-SCID patients suffer from lymphopenia, severely impaired cellular and humoral immunity, failure to thrive, and recurrent infections. Currently available therapeutic options for this otherwise fatal disorder include bone marrow transplantation (BMT), enzyme replacement therapy with bovine ADA (PEG-ADA), or hematopoietic stem cell gene therapy (HSC-GT). Although varying degrees of immune reconstitution can be achieved by these treatments, breakdown of tolerance is a major concern in ADA-SCID. Immune dysregulation such as autoimmune hypothyroidism, diabetes mellitus, hemolytic anemia, and immune thrombocytopenia are frequently observed in milder forms of the disease. However, several reports document similar complications also in patients on long-term PEG-ADA and after BMT or GT treatment. A skewed repertoire and decreased immune functions have been implicated in autoimmunity observed in certain B-cell and/or T-cell immunodeficiencies, but it remains unclear to what extent specific mechanisms of tolerance are affected in ADA deficiency. Herein we provide an overview about ADA-SCID and the autoimmune manifestations reported in these patients before and after treatment. We also assess the value of the ADA-deficient mouse model as a useful tool to study both immune and metabolic disease mechanisms. With focus on regulatory T- and B-cells we discuss the lymphocyte subpopulations particularly prone to contribute to the loss of self-tolerance and onset of autoimmunity in ADA deficiency. Moreover we address which aspects of immune dysregulation are specifically related to alterations in purine metabolism caused by the lack of ADA and the subsequent accumulation of metabolites with immunomodulatory properties.

## The ADA Metabolism

### The ADA enzyme

**Figure 1:**
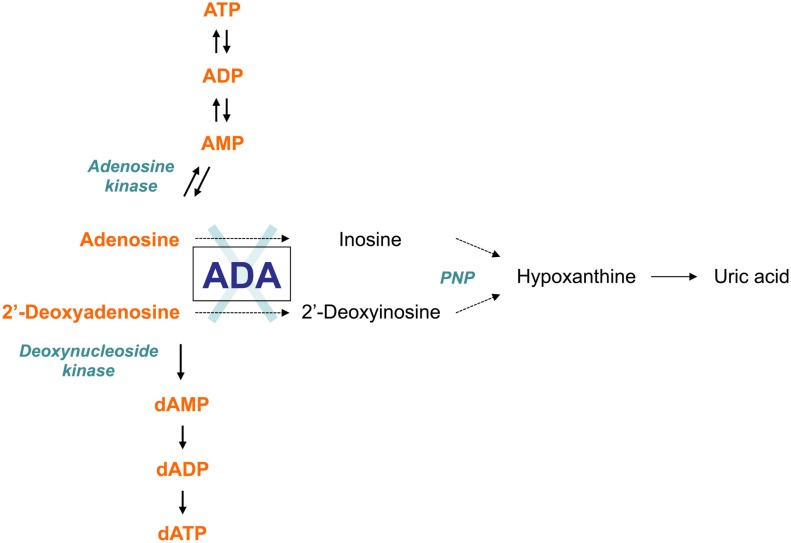
**The adenosine deaminase (ADA) metabolism**. ADA is an enzyme of the purine salvage pathway, which catalyzes the irreversible deamination of adenosine and 2′-deoxyadenosine into inosine and 2′-deoxyinosine, respectively. Most adenosine derives from endogenous breakdown of ATP and degradation of RNA, or is taken up exogenously by ubiquitously expressed nucleoside transporters. Unlike adenosine, 2′-deoxyadenosine is formed by DNA degradation is predominantly catabolized by ADA. Further conversion of inosine nucleoside leads to hypoxanthine, which can either enter a non-reversible pathway to uric acid or salvaged back into other mononucleosides. In the absence of ADA, the presence of these alternative “bypass” pathways results in normal concentrations of the catabolic products of the enzyme reaction in patients with ADA-SCID. Conversely, the levels of ADA substrates, adenosine and 2′-deoxyadenosine, are not only found in increased amounts in extracellular body fluids, but they also “spill over” into additional pathways normally only minimally utilized, thus contributing to the pathogenic mechanisms of the disease.

As an enzyme of the purine salvage pathway, adenosine deaminase (ADA) catalyzes the deamination of adenosine and 2′-deoxyadenosine, as well as several naturally occurring methylated adenosine compounds (Hirschhorn and Ratech, 1980; Ratech et al., 1989). The deamination of adenosine and 2′-deoxyadenosine gives rise to inosine and deoxyinosine, respectively (Hirschhorn and Candotti, 2006). Further conversion of these deaminated nucleosides leads to hypoxanthine, which can be either transformed irreversibly into uric acid or salvaged into mononucleosides (Figure 1).

Although ADA is present in all cell types, its enzyme activity differs considerably among tissues. The highest amounts in humans are found in lymphoid tissues, particularly the thymus, the brain, and gastrointestinal tract. The ADA enzyme is ubiquitously expressed both intracellularly and on the cell surface where it complexes with two molecules of CD26 as a combined protein (Kameoka et al., 1993).

### The ADA substrates adenosine and 2′-deoxyadenosine

2′-Deoxyadenosine is a component of DNA and primarily derives from its breakdown. Therefore, 2′-deoxyadenosine concentration is expected to be highest at sites of cell death, such as the bone marrow and thymus, where lymphocytes undergo apoptotic death during differentiation and selection. 2′-Deoxyadenosine behaves as a cytotoxic metabolite and is generally considered the primary cause of lymphotoxicity in ADA-severe combined immunodeficiency (SCID; Hirschhorn and Candotti, 2006). The most striking metabolic alteration in ADA deficiency is the accumulation of massive amounts of dATP in erythrocytes and lymphocytes (Hirschhorn et al., 1992). This results from uptake of increasing 2′-deoxyadenosine present in surrounding body fluids with subsequent intracellular phosphorylation and trapping.

Adenosine on the other hand is a component of adenine nucleotides including ATP and RNA (Hirschhorn and Candotti, 2006). Elevated adenosine levels, as occurring in ADA deficiency contribute to apoptosis and block in the differentiation of thymocytes, causing severe T lymphopenia in mice and humans (Apasov et al., 2001; Gaspar et al., 2009; Poliani et al., 2009). Moreover adenosine, acting through cell surface G protein-coupled receptors, functions as an extracellular signal transducer in a variety of physiological processes (Olah and Stiles, 1995). Apart from T-cell receptor signaling (Huang et al., 1997), adenosine is involved in the control of heart rate and blood pressure (Fukunaga et al., 1982; Belardinelli et al., 1989), renal function (Churchill and Bidani, 1982), inflammatory responses (Blackburn, 2003), and in neurotransmission (Fredholm and Dunwiddie, 1988).

## ADA-SCID

Adenosine deaminase deficiency is the second-most prevalent form (approximately 20%) of SCID. The overall incidence in Europe is estimated to range between 1:375,000 and 1:660,000 live births. ADA-deficient patients suffer from lymphopenia, severely impaired cellular and humoral immune function, failure to thrive, and a rapidly fatal course due to infection (Hirschhorn and Candotti, 2006). Moreover, autoimmune manifestations are commonly observed in milder forms of the disease. Currently available therapeutic options include bone marrow transplantation (BMT), enzyme replacement therapy with bovine ADA (PEG-ADA), or hematopoietic stem cell gene therapy (HSC-GT).

### Immune defects

Lymphopenia and attrition of immune function over time are the two findings common to all presentations of ADA deficiency. It is associated with thymic hypoplasia and a severe depletion of all three major categories of lymphocytes, T-, B-, and NK-cells (Buckley et al., 1997). Absence of cellular and humoral immunity and a rapidly fatal course due to infections with fungal, viral, and opportunistic agents are characteristic of early onset forms of ADA deficiency (Giblett et al., 1972; Buckley et al., 1997). Total immunoglobulin levels may be only slightly depressed at birth due to the maternal contribution of IgG, whereas both IgM and IgA, which ordinarily do not cross the placental barrier, are often absent. However, once IgG levels decline as maternal antibodies are cleared, a pronounced hypogammaglobulinemia signals the absence of humoral immunity (Morgan et al., 1987; Hirschhorn and Candotti, 2006). About 20% of ADA-SCID cases occur later in childhood (delayed) or beyond (late/adult onset). Delayed or late-onset patients have significant immunodeficiency, but variable clinical manifestations (Ozsahin et al., 1997). These forms show progressive immunological and clinical deterioration, often associated with autoimmune manifestations, including hemolytic anemia, and immune thrombocytopenia (Parkman et al., 1975; Aiuti et al., 2003). Serum immunoglobulin levels are altered in late-onset patients, with IgG2 levels being highly reduced or absent. IgE levels are elevated and often associated to eczema and asthma. An inability to produce antibodies against polysaccharide and pneumococcal antigens was frequently found in ADA-SCID patients with milder forms of the disease (Levy et al., 1988).

### Non-immune defects

The initial and most devastating presentation of ADA-SCID is due to the immune defects (Gaspar et al., 2009). Nonetheless, several non-immune abnormalities have been described in ADA deficiency, indicating that this disease should be considered a systemic metabolic disorder (Aiuti et al., 2003; Hirschhorn and Candotti, 2006). ADA is ubiquitously expressed in all cell types; when absent, the systemic metabolic toxicity is frequently associated with organ damage (Sauer and Aiuti, 2009). These include hepatic and renal disease (Bollinger et al., 1996), skeletal alterations (Sauer et al., 2009), neurological abnormalities (Honig et al., 2007; Titman et al., 2008), and behavioral impairments (Rogers et al., 2001). Because complications from infections usually predominate in the clinical presentation of infants with ADA deficiency, the full spectrum of non-immunologic manifestations and their natural course may be obscured (Honig et al., 2007). It is important to note, that several abnormalities have been described in few patients only, and might reflect effects of infectious agents rather than primary defects due to ADA deficiency: i.e., renal and adrenal abnormalities, phyloric stenosis, and hepatic disease (Hirschhorn and Candotti, 2006).

## Therapies for ADA Deficiency

**Figure 2:**
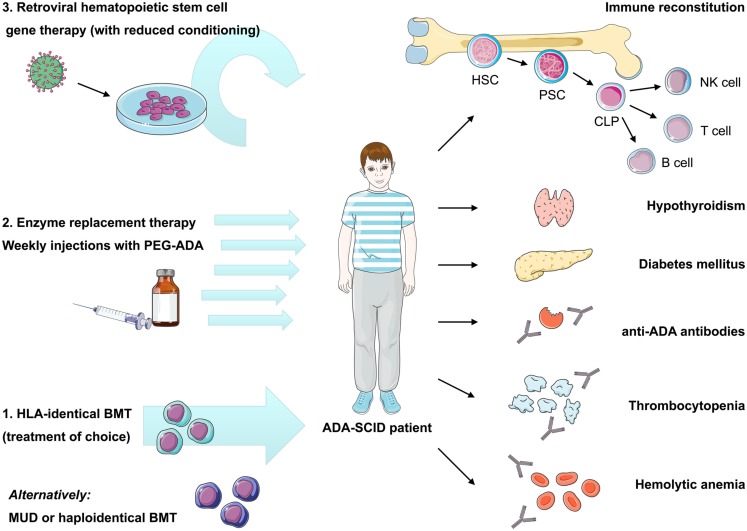
**Current therapeutic options in ADA-SCID and reported autoimmune manifestations after treatment**. Immune reconstitution in ADA deficiency can be achieved by bone marrow transplantation, enzyme replacement, or gene therapy, nonetheless recovery of immune functions may vary depending on the applied treatment and patient’s characteristics. Treatment of choice remains bone marrow transplantation from an HLA-identical sibling donor, while transplants from alternative donors are associated with high morbidity and mortality. Enzyme replacement therapy using pegylated bovine ADA is a non-curative treatment requiring weekly intramuscular injections with PEG-ADA. ADA-SCID has been the pioneer disease for the development of human gene therapy. It is based on the reinfusion of autologous HSC transduced with a retroviral vector containing the *ADA* cDNA. Variable degrees of immune reconstitution can be achieved by these treatments, but onset of autoimmunity is of concern in post-treatment ADA-SCID patients. Reported autoimmune manifestations include: autoimmune hypothyroidism, diabetes mellitus, thrombocytopenia, hemolytic anemia, and development of anti-ADA antibodies. HLA, human leukocyte antigen; BMT, bone marrow transplantation; MUD, matched unrelated donor; PEG-ADA, pegylated bovine ADA; HSC, hematopoietic stem cell; PSC, pluripotent stem cell; CLP, committed lymphocyte precursor; NK, natural killer cell.

Bone marrow transplantation with allogeneic HSC has long been considered the mainstay of ADA-SCID treatment. However, unlike other SCID forms, two other treatment options are available for ADA-SCID: enzyme replacement therapy with pegylated bovine ADA (PEG-ADA) and autologous HSC-GT (Hershfield et al., 1987; Aiuti et al., 2009). The availability of different treatment modalities presents an opportunity for improved patient care but also difficulties in deciding upon the specific choice of treatment for individual patients (Figure 2). Making the correct choice is further complicated by the fact that ADA deficiency is not purely an immune defect, and that the systemic manifestations, which can be of major clinical consequence, must also be managed (Gaspar et al., 2009).

### Hematopoietic stem cell transplantation

Hematopoietic stem cell-transplantation (BMT) from allogeneic human leukocyte antigen (HLA)-compatible sibling donors resulting in long-term survival and effective immune reconstitution is the treatment of choice for patients with ADA-SCID and other severe variants of primary immunodeficiencies. Since less than 20% of ADA-SCID patients have access to HLA-matched family donors, transplants are often performed from mismatched family or matched unrelated donors (Antoine et al., 2003; Gaspar et al., 2009; Ferrua et al., 2010). A recent retrospective analysis on the specific outcome of transplants for ADA-SCID collected data from several multicenter studies and analyzed the survival of 106 patients who received a total of 119 transplants (Hassan et al., 2012). BMT from matched sibling and family donors had a significantly better overall survival (86 and 81%) in comparison to BMT from matched unrelated (66%) and haploidentical donors (43%). Indicating that despite recent progress in transplantation, the use of alternative donors is still associated with a reduced overall survival (Gaspar et al., 2009). This is further complicated by the fact that ADA-SCID patients are more difficult to transplant especially from unrelated and haploidentical donors possibly due to their need for conditioning and the underlying metabolic nature of the disease (Gaspar et al., 2009; Sauer et al., 2009). While superior survival was seen in patients who received unconditioned transplants in comparison to myeloablative procedures (81 and 54%), non-engraftment was a major problem after unconditioned haploidentical transplants (Hassan et al., 2012).

Long-term immune recovery showed that regardless of transplant type, overall T-cell numbers were similar although a faster rate of T-cell recovery was observed following matched sibling or matched unrelated BMT. Humoral immunity and donor B cell engraftment was achieved in nearly all evaluable surviving patients and most patients were able to discontinue immunoglobulin replacement, suggesting that immune recovery is relatively complete (Hassan et al., 2012). According to the available data, the immunological and metabolic recovery after transplant is well maintained even after 10 years or longer in some patients (Gaspar et al., 2009).

Nevertheless delayed or suboptimal immune reconstitution as a result of poor early engraftment or gradual decline in immune functions is observed in a significant fraction of surviving patients (Gaspar et al., 2009). Complications such as graft-versus-host disease, autoimmune and inflammatory manifestations, persistent infections, and disease-related issues have been described (Honig et al., 2007; Titman et al., 2008; Mazzolari et al., 2009).

In summary, the results obtained with transplantation from HLA-identical siblings or family donors indicate superior donor/host compatibility and outcome both in terms of survival and sustained immune recovery. Whereas the current evidence suggests that haploidentical donor transplants (performed with or without conditioning) have a poor chance of success and are therefore only undertaken if no other treatment options are available (Gaspar et al., 2009).

### Enzyme replacement therapy with PEG-ADA

Enzyme replacement therapy with PEG-ADA was developed as lifesaving, not curative treatment for patients lacking an HLA-compatible donor. Attachment of PEG through lysine residues confers several therapeutically beneficial properties to ADA (Abuchowski et al., 1977; Davis et al., 1981). This chemical modification of the bovine enzyme reduces its immunogenicity and prevents its degradation by plasmatic proteases as well as the binding of neutralizing antibodies (Abuchowski et al., 1977; Davis et al., 1981). Thereby the circulating life of the compound is prolonged from minutes to days as clearance from the circulation is inhibited (Booth and Gaspar, 2009). Cellular uptake of PEG-ADA is insignificant and its distribution is limited to the plasma. Enzymatically active ADA continuously circulates and eliminates accumulating adenosine and 2′-deoxyadenosine metabolites (Chan et al., 2005). The principle of exogenous PEG-ADA administration is based on the direct conversion of accumulating ADA substrates in the plasma and the indirect reduction of intracellular toxic metabolites by diffusion.

**Figure 3:**
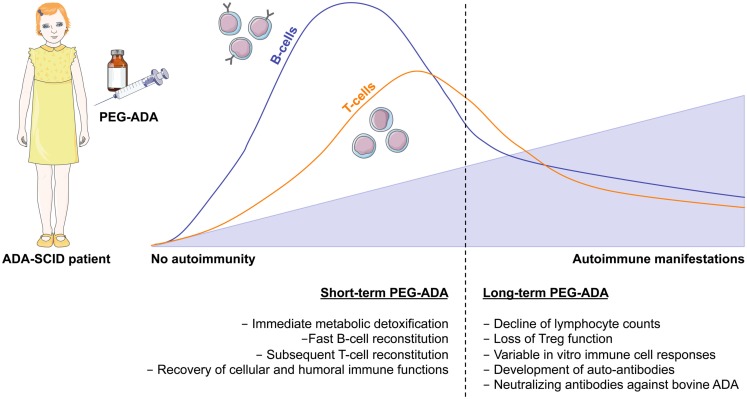
**Immune reconstitution and development of autoimmunity after PEG-ADA treatment**. Enzyme replacement therapy with pegylated bovine ADA is a lifesaving but non-curative treatment for ADA-SCID patients. It provides metabolic detoxification and protective immune function with patients remaining clinically well, but immune reconstitution is often suboptimal and may not be long-lived. Shortly after initiation of PEG-ADA treatment, patients show recovery of B-cell counts, followed by a gradual increase in T-cell numbers and reconstitution of immune cell functions. However, the long-term consequences of PEG-ADA treatment are unknown. Immune recovery in B and T- cells is below normal levels. Major concerns are the susceptibility to opportunistic infections and the development of autoimmunity due to lymphopenia with gradual decline of immune functions and perturbation of T- and B-cell tolerance.

To date more than 150 patients worldwide have received this treatment (Booth and Gaspar, 2009; Gaspar et al., 2009). PEG-ADA is usually administered weekly or bi-weekly by intramuscular injections throughout life. In general, PEG-ADA treatment seems to be well tolerated, with clinical benefits appreciable after the first month of therapy (Figure 3). Studies have shown that upon the initiation of PEG-ADA therapy, the absolute numbers of circulating T- and B-lymphocytes and NK-cells increase and protective immune function develops (Weinberg et al., 1993). Although only limited information is available, some analysis indicated that about half of PEG-ADA treated patients discontinued IVIg (Gaspar et al., 2009), whereas long-term follow-up suggests that immune recovery is often incomplete (Booth and Gaspar, 2009). Two retrospective studies showed that despite initial improvements, the lymphocyte counts of all PEG-ADA treated patients were below the normal range at all times. A gradual decline of mitogenic proliferative responses occurred after a few years of treatment and normal antigenic responses occurred less than expected (Kohn, 2008; Serana et al., 2010). No toxic or hypersensitivity reactions have been reported with PEG-ADA administration. However, several other side effects have been reported including manifestations of immune dysregulations including autoimmunity (type I diabetes, hypothyroidism, immune thrombocytopenia, hemolytic anemia) and allergic manifestations (Notarangelo et al., 1992; Ozsahin et al., 1997). An additional concern with PEG-ADA beyond about 8–10 years is the emergence of serious complications, including lymphoid and hepatic malignancies, and progression of chronic pulmonary insufficiency (Gaspar et al., 2009).

The main side effect associated with the use of PEG-ADA is the development of anti-ADA antibody. The development of specific IgG antibody to bovine peptide epitopes of PEG-ADA has been reported by several groups and often coincides with an improvement in humoral immunity (Chaffee et al., 1992; Lainka et al., 2005; Booth et al., 2007). In about 10% of treated patients, inhibitory antibodies lead to the enhanced clearance of PEG-ADA with subsequent decline in metabolic parameters and immune function (Chaffee et al., 1992; Hershfield, 1995; Lainka et al., 2005).

### Gene therapy

Hematopoietic stem cell gene therapy is a promising therapeutic option for genetic disorders of the immune system (Bordignon and Roncarolo, 2002; Fischer et al., 2005). ADA-deficient SCID has been under intensive preclinical and clinical investigation and nowadays represents a paradigmatic model of gene therapy for inherited disorders (Aiuti et al., 2003, 2007). The strong rationale for somatic gene therapy and the need for alternative treatments led to the design of clinical trials based on retroviral-mediated gene transfer of the normal *ADA* gene into autologous HSCs (Aiuti, 2004). Replication-deficient, recombinant retroviruses derived from the backbone of Moloney murine leukemia virus (MLV) were selected for these trials because of the available long-term experience and their ability to efficiently insert the therapeutic gene into the genome of dividing hematopoietic cells.

Since 2000, 37 patients have been treated in Italy, UK, and USA, achieving substantial clinical benefit in the majority of them. All patients received reduced intensity conditioning and PEG-ADA was discontinued to exploit the selective growth advantage for gene corrected over defective cells. At present, all patients are alive and in 26 patients PEG-ADA is no longer required (Aiuti et al., 2009; Gaspar et al., 2011; Montiel-Equihua et al., 2012). Gene therapy resulted in sustained engraftment of transduced cells, increased lymphocyte counts, improvement of cellular and humoral responses, and effective metabolic detoxification (Aiuti et al., 2009; Gaspar et al., 2011). Gene corrected cells were detected in myeloid and lymphoid subsets, the latter being more represented due to their survival advantage (Aiuti et al., 2009; Gaspar et al., 2011). In the HSR-TIGET study, all children maintained stable engraftment of vector ADA-transduced CD34+ cells with sustained systemic detoxification (Aiuti et al., 2009). At present, 15 of the 18 treated children do not require enzyme replacement therapy, with the longest follow-up at 11 years after treatment (Aiuti et al., 2009; Ferrua et al., 2010). These findings demonstrated the clinical efficacy of *ADA* gene transfer in restoring normal immune function and metabolic functions of ADA-SCID patients.

Unlike trials with gammaretroviral vectors in other diseases like X-linked SCID (Hacein-Bey-Abina et al., 2008; Howe et al., 2008), chronic granulomatous disease (Ott et al., 2006) and Wiskott–Aldrich Syndrome (Trobridge, 2011), the cumulative experience of these studies for ADA-SCID (Aiuti et al., 2009; Ferrua et al., 2010; Montiel-Equihua et al., 2012) did not reveal leukemic or oncogenic events, indicating that ADA-SCID gene therapy has a favorable risk/benefit profile. Unique risk factors may have contributed to the differential outcome of the other trials, such as vector constructs or promoters, inappropriate expression of transgenes involved in cell signaling (Kohn, 2008), cooperation between transgene and cellular oncogenes (Dave et al., 2009), or the disease background itself (Shou et al., 2006).

## Autoimmunity in ADA-SCID

Immunodeficiency and autoimmune phenomena may occur concomitantly in the same individual (Etzioni, 2003). Immune dysregulation, which often manifests as multiple forms of autoimmunity, can affect both the adaptive and innate immune system, indicating that all these immune components are required for the appropriate development of tolerance in humans (Cunningham-Rundles, 2011). Since varying degrees of immune reconstitution can be achieved by the available treatment options for ADA-SCID, breakdown of tolerance and development of autoimmunity can represent a major concern. Autoimmune dysregulation are frequently observed in patients with milder forms of the disease or late-onset patients. They may manifest as autoimmune hypothyroidism, diabetes mellitus, hemolytic anemia, and immune thrombocytopenia (Notarangelo et al., 1992; Ozsahin et al., 1997; Figure 2).

Similar complications, such as autoimmune hemolytic anemia and autoimmune thyroiditis, have also been reported in at least nine patients after long-term PEG-ADA treatment (Ratech et al., 1989; Notarangelo et al., 1992; Ozsahin et al., 1997; Gaspar et al., 2009; Serana et al., 2010). Refractory hemolytic anemia was fatal in three patients (Gaspar et al., 2009). Two additional studies assessed defects in the lymphoid compartments of ADA-SCID patients following PEG-ADA. Different degrees of abnormalities in the B-cell compartment and inability to respond to vaccines, despite the presence of normal serum-Ig or hypogammaglobulinemia were reported (Malacarne et al., 2005). Moreover, a retrospective longitudinal analysis in ADA-SCID patients treated with PEG-ADA showed that decreased levels of newly produced B cells underlie the progressive and significant decrease in circulating B cells in these patients (Serana et al., 2010). Since long-term PEG-ADA treatment is associated with abnormalities in B cell subsets, but often also with a decrease in T-cell functions (Malacarne et al., 2005), a limited B or T-cell repertoire combined with alterations in peripheral tolerance could further favor breakdown of tolerance (Figure 3).

No specific reports on immune dysregulation or autoimmunity in BMT-treated ADA-SCID patients are available in literature (Serana et al., 2010). Nevertheless, autoimmune manifestations have been reported in larger single-center studies on BMT-treated patients with various kinds of immunodeficiencies, including ADA deficiency (Mazzolari et al., 2009; Neven et al., 2009). The major immune dysregulations observed in both studies included thyroid autoimmunity, autoimmune hemolytic anemia, and glomerulonephritis (Mazzolari et al., 2009; Neven et al., 2009).

Most recently autoimmune manifestations have also been described in patients treated with HSC-GT (Aiuti et al., 2009). Four ADA-SCID patients, including one patient that already showed immune dysregulation while on PEG-ADA, developed signs of autoimmunity, such as hemolytic anemia, thrombocytopenia, autoimmune hepatitis, and autoimmune thyroiditis (Aiuti et al., 2009 and unpublished observation).

## ADA-Deficient Mouse Model

The availability of a genetic animal model for ADA deficiency allowed a wide range of biochemical and immunological experiments that are not feasible in humans. The first attempts to generate ADA-deficient mice lead to their perinatal death due to severe liver damage (Blackburn et al., 1998). Subsequent studies suggested that ADA expression in trophoblast cells of the placenta is critical for fetal development in the mouse. Thus, ADA-deficient mice were successfully generated by specifically targeting expression of an *ADA* minigene to the trophoblast lineage of ADA+/− mice and by inter-crossing these mice. This gave rise to litters that contained mice expressing the *ADA* minigene in their placenta that were also homozygous for the ADA null allele (ADA−/−; Blackburn et al., 1998).

### Untreated ADA−/− mice

The ADA−/− mouse reproduces not only the biochemical but also the immunological abnormalities of the human disease phenotype. They manifest both combined immunodeficiency as well as metabolic abnormalities and are therefore commonly used to assess the effect of ADA deficiency not only on the lymphoid organs and peripheral blood, but also its systemic organ toxicity. ADA−/− deficient mice die at approximately 3 weeks of age from severe respiratory distress (Blackburn et al., 1998).

Initial examinations of the thymus and spleen revealed a substantial decrease in organ size. The cellular proportion from the thymus of ADA−/− mice showed a significant increase in the percentage double-negative immature thymocytes, accompanied by a decrease in the percentage of CD4+ or CD8+ single-positive thymocytes. T-cell apoptosis was abundant in the ADA-deficient thymi (Blackburn et al., 1998). ADA−/− splenic B lymphocytes showed defects in proliferation and activation with high propensity to undergo B cell receptor-mediated apoptosis. As a result, profound loss of germinal center architecture was noted, which may be responsible for impaired B cell development (Aldrich et al., 2003). Lymphopenia was also seen in the peripheral circulation, confirming that this model of ADA deficiency exhibits a SCID phenotype.

At death, the severe immune deficiency and organ alterations are the most prominent features, whereas no apparent autoimmune manifestations can be observed. The almost complete absence of effector T- and B-cell populations in these mice and the high levels of anti-inflammatory adenosine might prevent their development in the first 3 weeks of life. Reconstitution of effector T- and B-cells as well as metabolic detoxification after treatment might therefore be requirements for the onset of autoimmunity (Sauer et al., 2012a).

### Model for autoimmunity in ADA-deficient mice

Similarly to ADA-SCID patients, ADA−/− mice can be treated with PEG-ADA, HSC-GT with transduced BM ADA−/− cells, or BMT with wild type donor cells (Mortellaro et al., 2006; Sauer et al., 2009). A dose of 1000 U/kg/week of PEG-ADA starting from postnatal day 10 provides rescue and metabolic detoxification in ADA−/− mice (Blackburn et al., 2000). HSC-GT is performed using a SIN-lentiviral vector driving ADA expression from the phosphoglycerate kinase (PGK) promoter (Mortellaro et al., 2006), instead of the gammaretroviral vector used in the clinical trial. A long-term comparative approach between these three treatment options revealed important new information on their efficacy and established a model for autoimmunity in the context of long-term PEG-ADA treatment (Sauer et al., 2012a).

The long-term survival of PEG-ADA, HSC-GT, and BMT-treated mice was comparable between the three groups (60–70% with respect to wildtype). This outcome was the result of an early mortality in the BMT and HSC-GT treated groups, while PEG-ADA treated mice had a less stable long-term survival. As expected from the fact that PEG-ADA remains in circulation without entering in cells, ADA activity in PEG-ADA treated mice was exclusively detectable in the plasma. Reconstitution of enzymatic activity in RBC, BM, spleen, and thymus from BMT-treated mice was comparable to wildtype, while only slightly lower in HSC-GT treated mice (Sauer et al., 2012a).

Strikingly, ADA−/− mice treated with PEG-ADA developed multiple autoantibodies and hypothyroidism in contrast to mice treated with HSC-GT or BMT. Proliferation of various lymphocyte subpopulations, including B cells and highly abnormal antibody production affecting all types of antibody subclasses was observed in PEG-ADA treated mice. Moreover, autoantibodies that reacted to ADA, platelets, the thyroid, and the gastrointestinal tract were detected in the sera from PEG-ADA treated mice. Focal atresia with non-secreting follicles, an increase in apoptotic cells in affected tissue areas and significantly elevated levels of thyroid-stimulating hormone (TSH) represent signs of autoimmune hypothyroidism. The role of autoantibodies against the stomach and intestine developing in PEG-ADA treated mice, without causing gross pathological alterations, remained unclear. However, it was hypothesized that the occurrence of antibody responses to GI tissues not only interferes with nutrient uptake, but also reflect alterations in gastrointestinal immunity (Sauer et al., 2012a). The established mouse model for autoimmunity after PEG-ADA treatment represents a valuable model for future studies on the *in vivo* effects of PEG-ADA on immune cell function and inflammatory responses.

Interestingly, PEG-ADA treated mice produced antibodies to ADA, platelets, the thyroid, and gastrointestinal tract, but not other organs such as the pancreas or endocrine glands. The strong overlap of autoimmune manifestations observed in this model of autoimmunity in ADA−/− mice with those reported in ADA-deficient patients suggests that a component of autoimmune susceptibility may map to the target tissue. In both humans and in mouse models, single genetic loci have been linked with susceptibility to multiple autoimmune diseases. The genes underlying such loci, including AIRE, FoxP3, CTLA-4, and PTPN22, are likely to confer a general predisposition to the failure of immune tolerance and development of an auto-aggressive immune response (Hill et al., 2007). However, other loci are clearly disease specific, and presumably modify a generalized predisposition to confer organ/disease specificity. Interestingly, recent studies have implicated ADA polymorphisms in the development of type1 diabetes and rheumatoid arthritis (Sebastiani et al., 2006; Saccucci et al., 2009).

## Role of ADA Metabolites in Immune Cell Development and Function

Although autoimmunity is frequently observed in certain immunodeficiencies, there is accumulating evidence that ADA deficiency predisposes to this phenomenon not only through general mechanisms of immune dysregulation but also through specific alterations caused by the accumulating ADA metabolites. Main feature of ADA deficiency is the gradual accumulation of adenosine and 2′-deoxyadenosine nucleosides. In the absence of ADA, these nucleosides are metabolized differently into AXP or dAXP, respectively, and exert distinct biochemical action (AXP: AMP, ADP, or ATP; dAXP: dAMP, dADP, or dATP). Several pathophysiological mechanisms have been proposed to describe the role of ADA substrates in cytotoxicity as well as their immunomodulatory properties in patients and in the ADA-deficient mouse model (Hirschhorn and Candotti, 2006). The major effects of adenosine, 2′-deoxyadenosine and their nucleotide byproducts are summarized in Table 1.

**Table 1:** **Effects of ADA metabolites on lymphocyte development and function**.

ADA metabolite	Cell type	Mouse/human	Mode of action	Reference
2′-Deoxyadenosine	Lymphocytes	Human	Inhibition of SAHH activity results in accumulation of *S*-adenosylhomocysteine and inhibition of transmethylation reactions	Hershfield et al. (1979) and Benveniste et al. (1995)
	Fibroblasts	Mouse	SAHH acts as a physiological modulator of Fas-mediated cell death	Ratter et al. (1996)
	T cells	Human	Inhibition of T-cell activation by aberrant Adora2a signaling and PKA hyperactivation	Cassani et al. (2008)
dATP	Lympocytes and RBC	Human	Intracellular ATP depletion	Siaw et al. (1980), Simmonds et al. (1982), Koller et al. (1984), and Simmonds et al. (1984)
	T cells	Human	Inhibition of ribonucleotide reductase causes an imbalance of dNTPs and an impairment of DNA synthesis	Waddell and Ullman (1983), Benveniste et al. (1995)
	T cells	Human	Accumulation of DNA single strand breaks	Cohen and Thompson (1986) and Gangi-Peterson et al. (1999)
	T cells	Mouse	Inhibition of thymocyte development past the CD4−/CD8− double-negative stage	Van de Wiele et al. (2002) and Van de Wiele et al. (2006)
	B cells	Human	Nucleotide pool imbalance affects TdT activity during V(D)J recombination in the bone marrow	Gangi-Peterson et al. (1999)
Adenosine	T cells	Human	Compromised TCR/CD28-driven proliferation and cytokine production, defective activation of NF-κB transcriptional events	Cassani et al. (2008)
	Resting T cells	Human	Upregulation of CD152, CTLA-4, normally involved in the termination of immune responses	Vendetti et al. (2002)
	T cells	Mouse	Decreased TCR-triggered activation and upregulation of activation markers	Apasov and Sitkovsky (1999), Apasov et al. (2001)
	Activated T cells	Mouse	Inhibition of IL-2, TNFα, and INFγ secretion	Erdmann et al. (2005) and Lappas et al. (2005)
	B cells	Human	Adora2a signaling interferes with BCR- and TLR-function, inhibition of B-cell activation after stimulation	Sauer et al. (2012b)
	B cells	Mouse	Profound loss of GC, susceptibility to apoptosis, defects in B-cell proliferation and activation, block in Ag-dependent B-cell maturation	Aldrich et al. (2003)
	B cells	Mouse	Increase of intracellular cAMP suppresses the activation of NF-κB after BCR and TLR-stimulation	Minguet et al. (2005)
	Tregs	Mouse/human	Adora signaling causes alterations in the CD39/CD73 adenosinergic machinery, upregulation of FoxP3	Sauer et al. (2012a)
	Adaptive Tregs	Mouse	Adora2a signaling inhibits the generation of adaptive effector T cells and promotes the induction of adaptive Tregs, upregulation of FoxP3	Zarek et al. (2008)
ATP	T cells	Human	Purinergic stimulation through P2X receptors prolongs TCR-initiated activation and IL-2 secretion	Yip et al. (2009)
	T cells	Mouse	Antagonism of P2X blunts TCR-mediated activation and results in unresponsiveness to subsequent stimulation	Schenk et al. (2008)
	Tregs	Mouse	Activation of P2X7 inhibits the suppressive potential and stability of Tregs	Schenk et al. (2011)

*Adora2a, adenosine 2a receptor; ATP, adenosine-5′-triphosphate; BCR, B cell receptor; cAMP, 3′,5′-cyclic adenosine monophosphate; dATP, 2′-deoxyadenosine 5′-triphosphate; dNTPs, deoxynucleotides; GC, germinal center; INFγ, interferon gamma; NF-κB, nuclear factor kappa B; P2X/P2X7, purinergic receptors; PKA, cAMP-dependent protein kinase A; RBC, red blood cells; SAHH, *S*-adenosylhomocysteine hydrolase; TCR, T-cell receptor; TdT, terminal deoxynucleotidyl transferase; TNFα, tumor necrosis factor alpha; Tregs, naturally occurring regulatory T cells*.

### Cytotoxicity of 2′-deoxyadenosine and dATP

Based upon *in vivo* and *in vitro* findings, several mechanisms are believed to account for the block of lymphocyte development in ADA-SCID (Hirschhorn and Candotti, 2006). The biochemical hallmarks of ADA deficiency consist of the general belief, that 2′-deoxyadenosine is the primary cause of lymphotoxicity in ADA-SCID, which exerts its effects at the nucleoside level or after conversion to dATP. Although 2′-deoxyadenosine is a weak substrate for adenosine kinase and deoxycytidine kinase, in the absence of ADA these enzymes can phosphorylate 2′-deoxyadenosine. In turn, the resulting dATP pool expansion may interfere with a number of critical metabolic pathways.

These ADA substrate accumulations inhibit methyl-transfer reactions by suicide inactivation of *S*-adenosylhomocysteine (SAH) hydrolase (Hershfield et al., 1979). dATP is known to be a feedback inhibitor of ribonucleotide reductase. Its inhibition causes an imbalance of deoxynucleotides (dNTP), leading to an impairment of DNA synthesis, which is critical for the expansion of lymphocytes in response to antigenic challenge (Benveniste et al., 1995).

### Role of adenosine as anti-inflammatory mediator

By binding to G-coupled adenosine receptors present on the surface of target cells, adenosine acts as an extracellular signal transducer to exert suppressive functions (Sitkovsky et al., 2004). Physiologically, adenosine-mediated triggering of these receptors can promote a fine-tuning of the inflammatory responses. In the context of defective ADA metabolizing enzyme, where the extracellular levels of adenosine are increased, this regulation may be exaggerated and cause immune dysfunction. This process is mostly regulated by the aberrant engagement of adenosine 2a receptor (Adora2a)-mediated signaling.

**Figure 4:**
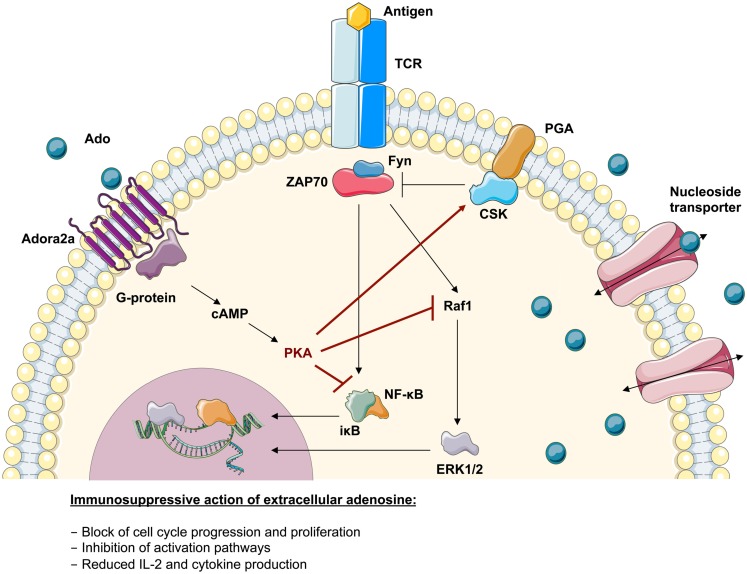
**Immunosuppressive action of adenosine in lymphoid cells**. Upon TCR-antigen-binding and T-cell activation, the translocation of activated transcription factors and the expression of anti-apoptotic factors supports their survival, proliferation, and differentiation. The adenosine 2a receptor (Adora2a) present on T-lymphocytes interacts with coupled G-proteins to stimulate cAMP formation. The effects of cAMP in T cells are almost entirely mediated by the cAMP-dependent protein kinase A (PKA). It has been shown that the inhibitory properties of PKA are mediated via activation of Csk or inhibition of Raf1. Consistently it has been shown that the severely compromised effector functions in ADA-deficient T cells associates with an intrinsically reduced ERK1/2 signaling, a decreased phosphorylation of IκB and an altered nuclear recruitment of NF-κB. This suggests that the accumulating adenosine levels in an ADA-deficient environment can directly antagonize biochemical events arising from TCR engagement.

Functional studies on T cells from ADA-deficient mice and patients showed an increased susceptibility to apoptosis as well as altered intra- and extracellular signaling leading to impaired T-cell function (Apasov and Sitkovsky, 1999; Apasov et al., 2001; Cassani et al., 2008). As summarized in Figure 4, the TCR-dependent activation defect in ADA deficiency is augmented by the immunosuppression through extracellular adenosine receptor triggering. Extracellular adenosine induces increased levels of cAMP in T-lymphocytes, which inhibits both proximal signaling events after TCR triggering as well as other downstream effector functions (Huang et al., 1997; Lappas et al., 2005; Ohta et al., 2009). In accordance with previous data obtained in thymocytes from ADA−/− mice (Apasov et al., 2001), IκBα phosphorylation after TCR triggering was low or undetectable in ADA-deficient cells (Cassani et al., 2008). Reduced levels of IκBα phosphorylation and degradation leads to low levels of NF-κB translocation and transcription of target genes in the nucleus, thereby contributing to the functional impairments of ADA-deficient T cells.

Less information is available about the effects exerted by adenosine on B-cell function. Similarly to the alterations in T cells described above, adenosine receptor ligation in B cells inhibits downstream responses to antigen receptor engagement like BCR-induced IκB phosphorylation and the NF-κB pathway after BCR or TLR4 stimulation (Minguet et al., 2005). Adenosine may thereby drive BCR-stimulated B cells toward an anergic rather than an immunogenic response. Recent findings showing defects in BCR and TLR signaling as well as in tolerance checkpoint control in human B cells are discussed and illustrated separately in section “Defects in B-cell tolerance in ADA-SCID.”

Overall, these evidences strongly suggest that rather than controlling a single pathway downstream of the TCR or BCR, the immune defects in ADA-deficient lymphocytes may involve multiple pathways converging toward the defective induction of lymphocyte activation. They also illustrate how extracellular adenosine levels can interfere with the downstream signaling transduction upon activation, thereby exerting its immunosuppressive activity on the transcriptional machinery. Because of cell-specific expression and regulation, aberrant adenosine receptor-mediated signaling might also contribute to the occurrence of autoimmune manifestations observed in some ADA-SCID patients (Kohn, 1996; Ozsahin et al., 1997).

### Role of ATP and other purinergic receptors

Stimulation of almost all mammalian cell types leads to the release of cellular ATP and autocrine feedback through a diverse array of purinergic receptors (Junger, 2011). ATP binds to two classes of purinergic P2 receptors in the plasma membrane of eukaryotic cells: P2X receptors, which are ligand-gated ion channels, and heterotrimeric G protein-coupled P2Y receptors (Schenk et al., 2011). Depending on the types of purinergic receptors that are involved, autocrine signaling can promote or inhibit immune cell activation and fine-tune adaptive immune responses (Junger, 2011; Schenk et al., 2011).

In addition to the autocrine feedback mechanisms that regulate the function of healthy immune cells, purinergic receptors allow immune cells to recognize ATP that is released from damaged or stressed host cells. Thus, the purinergic signaling systems of immune cells serve an important function in the recognition of danger signals. ATP that is released by stressed cells guides phagocytes to inflammatory sites and promotes clearance of damaged and apoptotic cells (Elliott et al., 2009; Junger, 2011).

To date, little information is available on alterations in the ATP-induced regulation of immune cells in ADA deficiency. It is reported that dATP accumulation in the absence of ADA leads to a cellular depletion of ATP (Siaw et al., 1980; Simmonds et al., 1982, 1984; Koller et al., 1984). The pool of extracellular ATP on the other hand might well be augmented in ADA-deficient lymphoid organs, due to the increased percentage of cells undergoing apoptosis. It can therefore be hypothesized that alterations in ATP concentrations in ADA deficiency also influence T-cell responses on the level of TCR induced activation and in response to stimuli from an inflammatory microenvironment.

## Break of Tolerance and Contribution of Lymphocytes to Autoimmunity in ADA Deficiency

Adaptive immunity requires sophisticated regulatory mechanisms to ensure protection to a variety of pathogenic microbes while maintaining immune self-tolerance and preventing autoimmunity (Sakaguchi et al., 2008). The main mechanisms for the induction and maintenance of a self-tolerant repertoire, which is diverse in antigen recognition, are central and peripheral tolerance. Central tolerance is the mechanism able to eliminate newly developing T cells and B cells that have high affinity to self (Mathis and Benoist, 2004). Central tolerance is distinct from peripheral tolerance in that it occurs while cells are still present in the primary lymphoid organs, whereas emigrant cells are controlled through peripheral tolerance mechanisms, after they reach the periphery (Wardemann and Nussenzweig, 2007; Klein et al., 2009). These include suppression of autoreactive cells by regulatory T cells and the generation of hyporesponsiveness (anergy) in lymphocytes, which encounter antigen in the absence of the co-stimulatory signals that accompany inflammation (Meffre and Wardemann, 2008).

Numerous mechanisms have been proposed to explain the break of tolerance and development of autoimmune manifestations, such as defective negative selection of autoreactive T-lymphocytes in the thymus, alterations in the number and/or function of regulatory T cells, defects of the central and peripheral B-cell tolerance checkpoints, impaired apoptosis of autoreactive lymphocytes, break of tolerance due to increased or decreased clearance of apoptotic cells and pathogens, or increased homeostatic lymphoid proliferation and cytokine secretion associated with lymphopenia (Carneiro-Sampaio and Coutinho, 2007; Westerberg et al., 2008; Notarangelo, 2009; Meffre, 2012).

### T-cell tolerance

Central T-cell tolerance mechanisms are based on the elimination or negative selection of the majority of T cells recognizing self with high affinity for negative selection in the thymus. Nonetheless thymic selection is not a tight process and T cells expressing low-avidity TCR on their cell surface are frequently released in the periphery, where they are potentially dangerous to the host as they can be effectively recruited into an autoimmune response (Parish and Heath, 2008).

A major cause of tolerance breakdown is associated with lymphopenia (Daikeler and Tyndall, 2007). This typical state of primary immunodeficiencies may contribute to the induction of spontaneous homeostatic proliferation of residual T cells allowing peripheral expansion of autoreactive cells with a skewed repertoire. Particularly, after conditioning or transplantation these cells may persist, since insufficient thymic reconstitution may affect the control of self-reactivity due to defective negative selection in the thymus and/or reduced regulatory T-cell development and function (Hauri-Hohl et al., 2007). In the case of ADA deficiency, it has been hypothesized that the structure and functions of the thymic microenvironment might be altered, either directly, by toxicity of purine metabolites, or indirectly, by failure of T cells arrested in their development to deliver supportive signals to the thymic stroma (Apasov et al., 2001).

Peripheral tolerance depends on the balance between immune responses to invading pathogens and immune tolerance to self-antigens. In the context of tissue damage and frequently occurring infections in primary immunodeficient patients, apoptotic cells represent a major source of autoantigen. Since apoptosis plays a major role in the deletion of autoreactive lymphocytes and the removal of virus-infected cells, defects in cell death have been implicated in the development of autoimmune diseases and persistent viral infection (Utz et al., 2000). The release of self-antigen into the intracellular space and their presentation mediated by dendritic cells or other antigen-presenting cells may prime naive autoreactive T cells, which were not eliminated by depletion or anergy (Waldner et al., 2004). Several mechanisms exist, including a spectrum of CD4+ regulatory T cells (Tregs), to suppress self-reactive T cells that escape thymic clonal deletion and attenuate anti-pathogen effector mechanisms from inducing immune pathology (Piccirillo and Thornton, 2004). There is ample evidence that Tregs actively mediate suppression to control immune responses to self- and non-self-antigens and the onset of autoimmunity (Bach, 2003; Sakaguchi, 2005). Lessons from other primary immunodeficiencies have provided unequivocal evidence for the essential role of Tregs in suppressing autoreactive T cells in the periphery (Westerberg et al., 2008). Rising of autoimmunity may not only be linked to a reduction in Treg numbers but also to attenuation of their suppressive activity (Sakaguchi et al., 2008). While this is principally mediated by cell–cell contact, recent findings revealed additional mechanisms of Treg-mediated suppression, including secretion of immunosuppressive cytokines, functional modification or killing of APC, and metabolic disruption (Vignali et al., 2008). Moreover, extracellular adenosine produced by Tregs, has been identified as one of the mechanisms mediating their suppressive activity (Sitkovsky et al., 2008; Mandapathil et al., 2010). Treg cells possess a unique biochemical signature amongst T cells in that they generate and sustain high adenosine concentrations. Since Tregs primarily mediate peripheral control of autoreactive T cells, it is conceivable that this compartment might be specifically affected in ADA-SCID (see also Defective Regulatory T Cell Function in ADA Deficiency). Consequently the autoimmune manifestations associated with ADA deficiency might be the result of an altered purine metabolism interfering with normal regulatory T-cell function (Sauer et al., 2012a).

### B-cell tolerance

A variety of mechanisms ranging from clonal deletion to functional inactivation by anergy of autoreactive B cells serve to shape the peripheral B-cell repertoire. Nevertheless, dysregulation of B cell development and autoantibody production is a characteristic of most autoimmune diseases including rheumatoid arthritis, systemic lupus erythematosus, and type 1 diabetes, but also immunodeficiencies such as CVID, Wiskott–Aldrich Syndrome, and X-linked agammaglobulinemia (Park et al., 2005; Cuss et al., 2006; Westerberg et al., 2008). In ADA deficiency, some of the observed immune dysregulation were hypothesized to be associated with a more restricted B-cell repertoire due to abnormalities in central B-cell generation or to a dysregulated expansion of these cells in the periphery.

Autoantibodies appear in the serum many years before the onset of clinical disease suggesting an early break in B-cell tolerance (Wardemann et al., 2003). Some of B-cell mediated autoimmune diseases, such as myasthenia gravis (MG), idiopathic autoimmune thrombocytopenic purpura (AITP), and Graves’ disease are characterized by auto-Abs production that destroy target tissues (Barsalou et al., 2011; Cunningham-Rundles, 2011). A remarkably high proportion of autoantibodies associated with systemic autoimmune diseases binds DNA, RNA, or macromolecular complexes that contain DNA or RNA. It has been hypothesized, that under certain circumstances these intracellular autoantigens become visible to the immune system when they accumulate during apoptosis. In fact the impaired clearance of apoptotic cell debris and dsDNA by macrophages might induce TLR signaling and differentiation of autoreactive B cells (Gaipl et al., 2006). Response to nucleic acid-containing immunecomplexes relies on the coengagement of endosomal members of the TLR family, TLR9 and TLR7 (Marshak-Rothstein, 2006). Therefore, self-antigens that can effectively engage both the BCR and either TLR7 or TLR9 might stimulate autoreactive B cells that are normally quiescent, through inherent adjuvant activity and trigger the development of systemic autoimmune disease (Marshak-Rothstein, 2006). In ADA deficiency, the metabolic basis underlying immune cell deficiency is the cytotoxic effect impact of the ADA substrates deoxyadenosine and dATP, leading to apoptosis of lymphocytes. It is therefore conceivable that developing B lymphocytes in affected lymphoid organs encounter massive amounts of nucleic acid. Nucleic acid-sensing TLRs might therefore represent Achilles’ heel in susceptible ADA-deficient patients by which relative tolerance for nucleic acid-containing antigens is breached and autoimmunity occurs (Kono et al., 2009).

## New Insights into Immune Cell Dysfunction and Onset of Autoimmunity in ADA Deficiency

Recent in-depth studies have revealed specific defects in ADA deficiency that may contribute to the onset of autoimmunity in these patients. Herein we discuss alterations in the adenosinergic machinery of ADA-deficient regulatory T cells and in B-cell tolerance in the absence of functional ADA.

### Defective regulatory T-cell function in ADA deficiency

**Figure 5:**
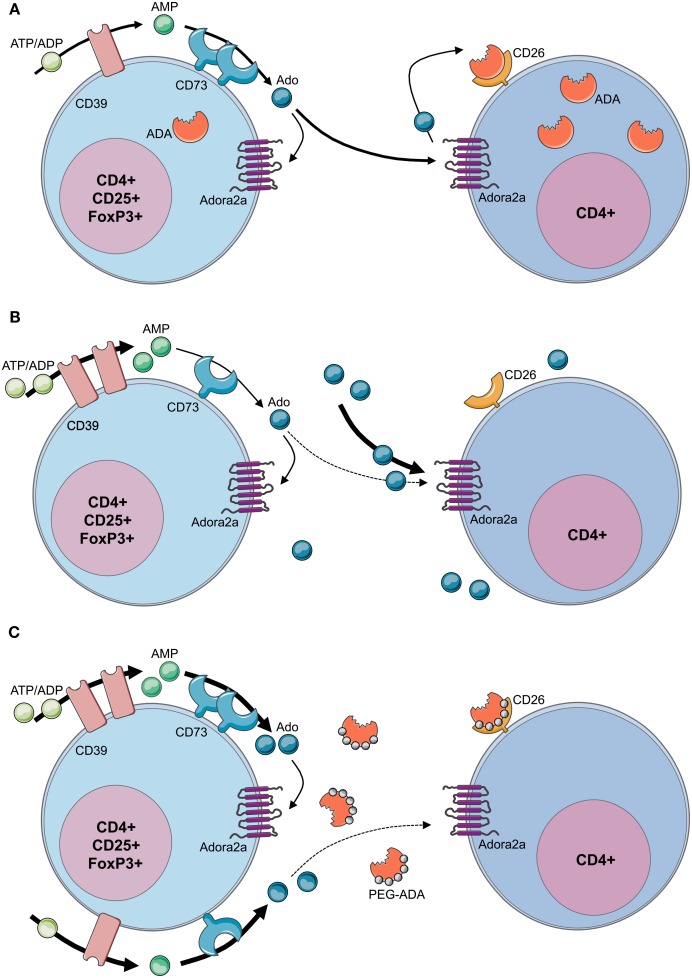
**Loss of regulatory T-cell function in ADA-SCID**. **(A)** By concomitant expression of CD39 and CD73, Tregs have the enzymatic machinery to generate and maintain high levels of extracellular adenosine. Contrarily to T effector cells, Tregs express low levels of ADA and CD26. Extracellular ATP or ADP is converted by the ectonucleotidase CD39 into AMP, which is further converted into adenosine by the CD73 ectoenzyme. The produced adenosine binds to the Adora2A receptor expressed on activated effector T cells, which are enriched in ADA and the surface-bound glycoprotein CD26. The coordinated expression of CD39 and CD73 on Tregs and Adora2a on T effector cells enables adenosine-mediated immunosuppression. **(B)** In the absence of ADA, deficient Tregs express high levels of CD39, increasing their capacity for ATP hydrolysis, but reduced levels of CD73 on their surface. Although both CD39 and CD73 are rate limiting for extracellular adenosine generation, CD73 is the last component of the ectoenzymatic chain. With accumulating adenosine levels, possibly to avoid a further increase of extracellular concentrations, CD73 is reduced and ADA−/− Tregs display a decreased suppressive activity toward T effector cells. Nevertheless no apparent autoimmune manifestations can be observed at onset, likely due to the severely reduced effector T- and B-cell populations. Moreover, the accumulating extracellular adenosine is likely to maintain an anti-inflammatory environment in these mice. **(C)** After PEG-ADA treatment, the adenosinergic machinery of CD39 and CD73 are upregulated, indicating an increased requirement for ATP hydrolysis and enhanced adenosine production. Despite the initial rescue of suppressive activity by upregulation of CD73 for elevated adenosine production, long-term PEG-ADA treatment interferes with Treg function by augmenting adenosine turnover. PEG-ADA present in the extracellular space eliminates adenosine produced by the ectoenzymatic chain and hinders adenosine-mediated suppression by interfering between adenosine and Adora2a expressed on T effector cells.

Although autoimmune manifestations are frequent findings in ADA-deficient patients with milder forms or in patients under PEG-ADA, mechanisms causing the loss of peripheral tolerance and onset of autoimmunity have remained elusive. CD4+CD25+FoxP3+ Tregs actively suppress pathological and physiological immune responses in order to maintain peripheral immune self-tolerance and prevent autoimmunity (Sakaguchi et al., 2008; Sitkovsky et al., 2008). Extracellular adenosine produced by Tregs has been described as one of the mechanisms mediating their suppressive activity (Figure 5A). Concordant expression of the ectoenzymes CD39 and CD73 has been reported both for murine and human Tregs (Borsellino et al., 2007; Deaglio et al., 2007; Mandapathil et al., 2010). The CD39 ectoenzyme produces AMP from ATP or ADP, which is subsequently converted into extracellular adenosine by the CD73 ectoenzyme (Hasko et al., 2008). Treg function requires the coordinated expression of the Adora2a on activated T effector cells to enable adenosine-mediated immunosuppression (Sitkovsky et al., 2008). Moreover, Tregs have been shown to express low levels of ADA, whereas T effector cells are enriched in ADA but express low levels of CD39 and CD73 (Mandapathil et al., 2010; Sauer et al., 2012a). This molecular profile of Tregs (CD39+CD73+ADA*low*) has functional importance, as it not only confers Tregs the capability to produce extracellular adenosine but also to sustain relatively high concentrations due to low ADA expression (Mandapathil et al., 2010).

Figure 5B summarizes recently described defects and functional alterations of the adenosinergic pathway in Tregs from ADA-deficient mice and patients (Sauer et al., 2012a). ADA−/− Tregs showed significantly higher expression of CD39, while expressing significantly less CD73. ADA−/− Tregs are sensitive to extracellular adenosine concentrations and the expression of CD73 is regulated by this metabolite. With adenosine accumulating in ADA−/− mice, possibly to avoid a further increase of extracellular concentrations, CD73 is reduced and ADA−/− Tregs display a decreased suppressive activity toward T effector cells. The underlying mechanism accounting for increased CD39 expression in ADA−/− Tregs remains to be elucidated. However, intracellular cAMP levels, which are elevated in the absence of ADA, have been reported to increase CD39 expression (Liao et al., 2010).

In order to dissect the cellular mechanisms leading to loss of peripheral tolerance, ADA−/− mice were studied after treatment with PEG-ADA, HSC-GT, and BMT. Although short-term PEG-ADA treatment initially rescued Treg-mediated suppression in comparison to untreated ADA−/− mice, their functionality became exhausted by long-term PEG-ADA treatment. Tregs from PEG-ADA treated animals maintained increased expression of CD39 and upregulated CD73 expression in comparison to age-matched wildtype controls. Consistently, CD39 activity measured by ATP consumption and AMP formation, as well as adenosine production by CD73 were significantly increased in comparison with wildtype Tregs. These results were confirmed in a cohort of patients including 7 PEG-ADA treated and 11 retroviral HSC-GT treated patients. The percentage of CD4+CD25+FOXP3+CD127−/low Tregs was significantly reduced in PEG-ADA treated patients and their expression of CD39 and CD73 ectonucleotidase were significantly increased. Unlike Tregs from HSC-GT treated patients and HD, Tregs isolated from PEG-ADA treated patients were unable to suppress the proliferation of effector cells (Sauer et al., 2012a).

The obtained results revealed an elevated adenosine catabolism in the presence of PEG-ADA, characterized by alterations in the adenosinergic machinery producing high levels of adenosine and a significantly increased turnover by the enzymatic activity of PEG-ADA. Upregulation of CD73 in treated ADA−/− mice and patients can therefore be interpreted as a compensatory mechanism representing a higher requirement for ATP/ADP to adenosine conversion in the presence of extracellular PEG-ADA. Despite the initial rescue of suppressive activity by upregulation of CD73 for elevated adenosine production, long-term PEG-ADA treatment interfered with Treg function by augmenting adenosine turnover. These findings fit the hypothesis that PEG-ADA present in the extracellular space eliminates adenosine produced by this ectoenzymatic chain and hinders adenosine-mediated suppression by interfering between adenosine and Adora2a expressed on T effector cells (Sauer et al., 2012a; Figure 5C).

### Defects in B-cell tolerance in ADA-SCID

Although PEG-ADA induces metabolic detoxification, BMT and HSC-GT provide superior restoration of purine metabolism and immune functions. However, it had remained unclear how patient’s B cells contribute to autoimmune complications and if B-cell tolerance is established properly in ADA-deficient patients before and after treatment.

**Figure 6:**
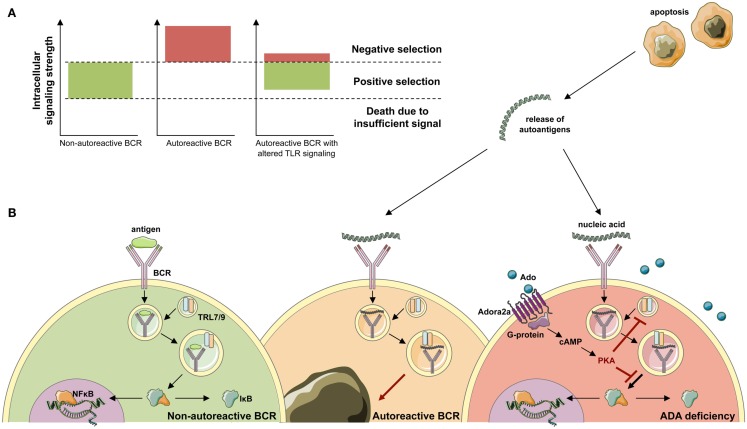
**Development of ANA-expressing B-cell clones in ADA deficiency**. **(A)** During B-cell development in the bone marrow, BCR signals provide a cell-intrinsic measure for negative or positive selection. By excessively weak or strong BCR signals, two thresholds identify functionally unfit or autoreactive B-cell clones. B cells that fail to rearrange and signal through their BCR die of neglect. Negative selection against autoreactive BCR specificities occurs following high avidity BCR interactions with antigen. While positive selection of B-cells requires persistent intermediate BCR signaling in both developing and mature B-cells. **(B)** Upon antigen-binding the BCR is internalized and transported to the cytoplasmic compartments containing Toll-like receptor 9 (TLR9) or TLR7. Physiological engagement of immunoreceptors leads to the phosphorylation and proteasomal degradation of IκB, thereby releasing NF-κB into the cytoplasm. Subsequently, NF-κB translocates to the nucleus and initiates the transcription of NF-κB target genes required for immune cell development and function. During the bone marrow differentiation of B cells, a major source of autoantigen is developing lymphocytes, which undergo apoptosis. Autoantigens are released in the extracellular space and form immune complexes. Binding of nucleic acid-containing immune complexes to an autoreactive BCR produces a strong activation signal through simultaneous activation of TLRs and leads to the depletion or receptor editing of the B cell. It can be hypothesized that in ADA deficiency this negative selection is dampened by adenosine present in the extracellular place and engagement of the Adora2a. Activation of Adora2a elevates intracellular cAMP through activation of adenylyl cyclase. In turn, cAMP activates PKA that blocks the BCR-induced phosphorylation of IκB to inhibit immunoreceptor-mediated NF-κB activation in the cytoplasm. The strong depletion signal coming from BCR and TLR coengagement is thereby lowered and ADA-deficient B cells carrying an autoreactive BCR egress into the periphery.

Random V(D)J recombination produces large numbers of antibodies displaying self-reactive specificities and during normal B-cell development the majority of these antibodies are removed at two distinct checkpoints in the bone marrow and periphery (Wardemann et al., 2003). Large numbers of self-reactive antibodies are removed from the B cell repertoire during the immature B cell stage in the bone marrow, where BCR-mediated selection plays a crucial role in controlling B-cell survival based on excessively strong or weak BCR signals that identify autoreactive or functionally unfit B cells (Goodnow, 1996; Nemazee et al., 2000; Cancro, 2009; Figure 6). Alterations of BCR signaling thresholds result in a defective central B-cell tolerance checkpoint and interfere with the removal of developing autoreactive B cells in humans (Ng et al., 2004; Menard et al., 2011). In addition to their BCRs, B cells also express TLRs that were originally described to bind microbial components but that are also able to recognize self-antigens (Marshak-Rothstein, 2006) and are involved in the removal of developing anti-nuclear antibody (ANA)-expressing B cells (Isnardi et al., 2008). Both BCR- and TLR-mediated B-cell responses have been reported to be modulated by adenosine receptor signaling and intracellular cyclic adenosine monophospate (cAMP), which are increased in ADA deficiency (Apasov et al., 2001; Hershfield, 2005; Minguet et al., 2005; Power Coombs et al., 2011).

B-cell tolerance checkpoints in ADA-SCID patients were assessed by cloning antibodies expressed by single B cells before and after successful HSC-GT (Sauer et al., 2012b). New emigrant/transitional and mature naïve B cells from ADA-deficient patients before HSC-GT contained high frequencies of autoreactive and ANA-expressing clones compared to healthy donors, revealing defective central and peripheral B-cell tolerance checkpoints in the absence of functional ADA.

The receptor candidates for the removal of ANA-expressing clones are nucleic acid-sensing endosomal members of the TLR family, TLR7 and TLR9 (Marshak-Rothstein, 2006), thereby suggesting that ADA impinges not only on BCR but more importantly TLR signaling. A similar mechanism has also been hypothesized to contribute to B-cell dysfunctions, defective B-cell proliferation, and activation observed in ADA-deficient mice (Aldrich et al., 2003; Hershfield, 2005). The accumulating adenosine blocks NF-κB activation in murine B cells stimulated through BCRs or TLR4 by LPS (Minguet et al., 2005; Power Coombs et al., 2011). In line with this hypothesis, we found that stimulation through TLR7 and TLR9 were significantly dependent on proper ADA enzymatic activity and adenosine receptor engagement, further underlining the inability of these receptors to function in the absence of functional ADA (Figure 6).

Strikingly, ADA-deficient patients treated with HSC-GT displayed quasi-normal early B-cell tolerance checkpoints as evidenced by restored efficient removal of developing autoreactive and anti-nuclear B cells. Hence, ADA plays an essential role in the establishment of early B-cell tolerance and the removal of developing autoreactive B cells in humans (Luning Prak, 2012; Sauer et al., 2012b).

## Concluding Remarks on the Occurrence of Autoimmunity after ADA-SCID Treatment

In summary, the available literature provides supporting evidence for a predisposition to autoimmunity in ADA deficiency. Alterations in both central and peripheral tolerance in T- and B-cells have been described to contribute to the pathogenesis of autoimmunity. Moreover, it is becoming increasingly clear that tolerance mechanisms and immune responses are specifically altered by the lack of ADA and the accumulation of its substrates.

Particularly, the impact of accumulating adenosine as anti-inflammatory mediator has to be underlined in ADA deficiency. The ligation of Adora2a receptors leads to an increase in cAMP levels, which in cooperation with PKA induces immunosuppression, attenuation of proximal signaling events after TCR and BCR triggering, and inhibition of downstream effector functions (Skalhegg et al., 1992; Huang et al., 1997; Lappas et al., 2005; Cassani et al., 2008; Sauer et al., 2012b). It can further be hypothesized that dampening of TCR- and BCR-downstream signaling interferes with the depletion signals during negative selection in central tolerance, thereby allowing the survival of autoreactive cells in ADA deficiency. In addition to its effects on T- and B-cells, adenosine is an important regulator, physiologically involved in inhibiting a variety of activated immune cells and in protecting tissues from acute inflammatory damage (Panther et al., 2003; Sitkovsky et al., 2004). Indeed we showed that Tregs require a balanced adenosine metabolism to exert their suppressive activity, since excessively high adenosine concentrations, or excessive conversion of extracellularly produced adenosine by PEG-ADA interferes with their suppressive function (Luning Prak, 2012; Sauer et al., 2012a).

The precise role of PEG-ADA alongside other treatment options is still undetermined, but it certainly allows rapid detoxification and stabilization of patients awaiting more definitive treatment (Booth and Gaspar, 2009). With a progressive loss of lymphocyte functions, the occurrence of neutralizing anti-ADA antibodies and autoimmune manifestations, long-term immunological reconstitution in PEG-ADA patients is often incomplete. It has been hypothesized that partial ADA correction resulting in low enzymatic activity may mimic late-onset patients, which typically display a higher prevalence of autoimmune manifestations (Ochs et al., 1992; Ozsahin et al., 1997; Luning Prak, 2012). Indeed, recent data underlined the importance of intracellular ADA expression and superior efficacy of gene therapy over PEG-ADA treatment for the development of functional T- and B-cell tolerance, including Tregs (Sauer et al., 2012a,b).

Both BMT and HSC-GT provide efficient reconstitution of the immune system through endogenous enzymatic ADA activity. BMT from an HLA-identical sibling donor remains the treatment of choice, while transplants from alternative donors are associated with high morbidity and mortality. The occurrence of mixed chimerism in transplanted patients with other primary immunodeficiencies is associated with a higher incidence of autoimmune manifestations (Moratto et al., 2011) and might well play a role also in ADA deficiency (Cancrini et al., 2010). Moreover, transplantation-induced lymphopenia is a possible cause for the homeostatic expansion of autoreactive T- and/or B-cells with subsequent loss of self-tolerance (Daikeler and Tyndall, 2007). Phenomena of immune dysregulation as occurring in the context of pre-transplant conditioning and BMT might further trigger the onset of autoimmunity (Etzioni, 2003).

In accordance with the current guidelines of the European group for Blood and Marrow Transplantation (EBMT) and given the long-term experience in gene therapy (Aiuti et al., 2009), this treatment can now be considered for all ADA-SCID patients lacking an HLA-identical sibling donor (Gaspar et al., 2009). After HSC-GT, high levels (50–90% on average) of gene correction were detected in T- and B- and NK-cells (Aiuti et al., 2009), leading to an efficient systemic detoxification and recovery of immune cell functions. However, as suggested by the cloning of single B-cell receptors, non-gene corrected cells may carry autoreactive specificities (Sauer et al., 2012b). It can be hypothesized that the co-existence of non-corrected autoreactive T- or B-cells and the restored functional T cell help could allow the development of autoimmune manifestations in ADA-SCID patients after HSC-GT (Aiuti et al., 2009). Modification of preparatory regimen or increased gene transfer efficiency by more robust approaches such as lentiviral vectors may further improve HSC-GT outcome for ADA deficiency (Mortellaro et al., 2006).

Adenosine deaminase-SCID remains a challenging condition to treat (Gaspar et al., 2009). With large-scale outcome studies still lacking, the choice between lifelong PEG-ADA, unrelated BMT and HSC-GT is currently based on the risk/benefit ratio, the availability, and costs of the three different treatment options (Gaspar et al., 2009). Due to the rarity of the disease and the small cohort numbers, accurate survey and long-term follow-up will be essential to determine the outcome following different treatments and their efficacy in restoring immune tolerance.

## Conflict of Interest Statement

The authors declare that the research was conducted in the absence of any commercial or financial relationships that could be construed as a potential conflict of interest.

## Abbreviations

ADA, adenosine deaminase; Adora, adenosine receptor(s); ANA, anti-nuclear antibody; BCR, B cell receptor; BMT, bone marrow transplantation; HLA, human leukocyte antigen; HSC, hematopoietic stem cell; HSC-GT, hematopoietic stem cell gene therapy; PEG-ADA, pegylated bovine ADA; SCID, severe combined immunodeficiency; TCR, T-cell receptor; TLR, Toll-like receptor; Tregs, naturally occurring regulatory T cells.

## References

[B1] Abuchowski A. van Es T. Palczuk N. C. Davis F. F. (1977). Alteration of immunological properties of bovine serum albumin by covalent attachment of polyethylene glycol. J. Biol. Chem. 252, 3578–3581 405385

[B2] Aiuti A. (2004). Gene therapy for adenosine-deaminase-deficient severe combined immunodeficiency. Best Pract. Res. Clin. Haematol. 17, 505–516 10.1016/j.beha.2004.05.012 15498720

[B3] Aiuti A. Cassani B. Andolfi G. Mirolo M. Biasco L. Recchia A. Urbinati F. Valacca C. Scaramuzza S. Aker M. Slavin S. Cazzola M. Sartori D. Ambrosi A. Di Serio C. Roncarolo M. G. Mavilio F. Bordignon C. (2007). Multilineage hematopoietic reconstitution without clonal selection in ADA-SCID patients treated with stem cell gene therapy. J. Clin. Invest. 117, 2233–2240 10.1172/JCI31666 17671653 PMC1934603

[B4] Aiuti A. Cattaneo F. Galimberti F. Benninghoff U. Cassani B. Callegaro L. Scaramuzza S. Andolfi G. Mirolo M. Brigida I. Tabucchi A. Carlucci F. Eibl M. Aker M. Slavin S. Al-Mousa H. Al Ghonaium A. Ferster A. Duppenthaler A. Notarangelo L. Wintergerst U. Buckley R. Bregni M. Valsecchi M. Rossi P. Ciceri F. Miniero R. Bordignon C. Roncarolo M. (2009). Long-term safety and efficacy of gene therapy for adenosine deaminase (ADA)-deficient severe combined immunodeficiency. N. Engl. J. Med. 360, 447–458 10.1056/NEJMoa0805817 19179314

[B5] Aiuti A. Ficara F. Cattaneo F. Bordignon C. Roncarolo M. G. (2003). Gene therapy for adenosine deaminase deficiency. Curr. Opin. Allergy Clin. Immunol. 3, 461–466 10.1097/00130832-200312000-00007 14612670

[B6] Aldrich M. B. Chen W. Blackburn M. R. Martinez-Valdez H. Datta S. K. Kellems R. E. (2003). Impaired germinal center maturation in adenosine deaminase deficiency. J. Immunol. 171, 5562–5570 14607964 10.4049/jimmunol.171.10.5562

[B7] Antoine C. Muller S. Cant A. Cavazzana-Calvo M. Veys P. Vossen J. Fasth A. Heilmann C. Wulffraat N. Seger R. Blanche S. Friedrich W. Abinun M. Davies G. Bredius R. Schulz A. Landais P. Fischer A. (2003). Long-term survival and transplantation of haemopoietic stem cells for immunodeficiencies: report of the European experience 1968–1999. Lancet 361, 553–560 10.1016/S0140-6736(03)12513-5 12598139

[B8] Apasov S. G. Blackburn M. R. Kellems R. E. Smith P. T. Sitkovsky M. V. (2001). Adenosine deaminase deficiency increases thymic apoptosis and causes defective T cell receptor signaling. J. Clin. Invest. 108, 131–141 10.1172/JCI10360 11435465 PMC209335

[B9] Apasov S. G. Sitkovsky M. V. (1999). The extracellular versus intracellular mechanisms of inhibition of TCR-triggered activation in thymocytes by adenosine under conditions of inhibited adenosine deaminase. Int. Immunol. 11, 179–189 10.1093/intimm/11.2.179 10069416

[B10] Bach J. F. (2003). Regulatory T cells under scrutiny. Nat. Rev. Immunol. 3, 189–198 10.1038/nri1026 12658267

[B11] Barsalou J. Saint-Cyr C. Drouin E. Le Deist F. Haddad E. (2011). High prevalence of primary immune deficiencies in children with autoimmune disorders. Clin. Exp. Rheumatol. 29, 125–130 21345299

[B12] Belardinelli L. Linden J. Berne R. M. (1989). The cardiac effects of adenosine. Prog. Cardiovasc. Dis. 32, 73–97 10.1016/0033-0620(89)90015-7 2664879

[B13] Benveniste P. Zhu W. Cohen A. (1995). Interference with thymocyte differentiation by an inhibitor of *S*-adenosylhomocysteine hydrolase. J. Immunol. 155, 536–544 7608534

[B14] Blackburn M. R. (2003). Too much of a good thing: adenosine overload in adenosine-deaminase-deficient mice. Trends Pharmacol. Sci. 24, 66–70 10.1016/S0165-6147(02)00045-7 12559769

[B15] Blackburn M. R. Aldrich M. Volmer J. B. Chen W. Zhong H. Kelly S. Hershfield M. S. Datta S. K. Kellems R. E. (2000). The use of enzyme therapy to regulate the metabolic and phenotypic consequences of adenosine deaminase deficiency in mice. Differential impact on pulmonary and immunologic abnormalities. J. Biol. Chem. 275, 32114–32121 10.1074/jbc.M005153200 10908569

[B16] Blackburn M. R. Datta S. K. Kellems R. E. (1998). Adenosine deaminase-deficient mice generated using a two-stage genetic engineering strategy exhibit a combined immunodeficiency. J. Biol. Chem. 273, 5093–5100 10.1074/jbc.273.9.5093 9478961

[B17] Bollinger M. E. Arredondo-Vega F. X. Santisteban I. Schwarz K. Hershfield M. S. Lederman H. M. (1996). Brief report: hepatic dysfunction as a complication of adenosine deaminase deficiency. N. Engl. J. Med. 334, 1367–1371 10.1056/NEJM199605233342104 8614422

[B18] Booth C. Gaspar H. B. (2009). Pegademase bovine (PEG-ADA) for the treatment of infants and children with severe combined immunodeficiency (SCID). Biologics 3, 349–358 19707420 PMC2726071

[B19] Booth C. Hershfield M. Notarangelo L. Buckley R. Hoenig M. Mahlaoui N. Cavazzana-Calvo M. Aiuti A. Gaspar H. B. (2007). Management options for adenosine deaminase deficiency; proceedings of the EBMT satellite workshop (Hamburg, March 2006). Clin. Immunol. 123, 139–147 10.1016/j.clim.2006.12.009 17300989

[B20] Bordignon C. Roncarolo M. G. (2002). Therapeutic applications for hematopoietic stem cell gene transfer. Nat. Immunol. 3, 318–321 10.1038/nrg784 11919567

[B21] Borsellino G. Kleinewietfeld M. Di Mitri D. Sternjak A. Diamantini A. Giometto R. Hopner S. Centonze D. Bernardi G. Dell’acqua M. L. Rossini P. M. Battistini L. Rotzschke O. Falk K. (2007). Expression of ectonucleotidase CD39 by Foxp3+ Treg cells: hydrolysis of extracellular ATP and immune suppression. Blood 110, 1225–1232 10.1182/blood-2006-12-064527 17449799

[B22] Buckley R. H. Schiff R. I. Schiff S. E. Markert M. L. Williams L. W. Harville T. O. Roberts J. L. Puck J. M. (1997). Human severe combined immunodeficiency: genetic, phenotypic, and functional diversity in one hundred eight infants. J. Pediatr. 130, 378–387 10.1016/S0022-3476(97)70199-9 9063412

[B23] Cancrini C. Ferrua F. Scarselli A. Brigida I. Romiti M. L. Barera G. Finocchi A. Roncarolo M. G. Caniglia M. Aiuti A. (2010). Role of reduced intensity conditioning in T-cell and B-cell immune reconstitution after HLA-identical bone marrow transplantation in ADA-SCID. Haematologica 95, 1778–1782 10.3324/haematol.2010.025098 20460637 PMC2948105

[B24] Cancro M. P. (2009). Signalling crosstalk in B cells: managing worth and need. Nat. Rev. Immunol. 9, 657–661 10.1038/nri2621 19704418 PMC2766863

[B25] Carneiro-Sampaio M. Coutinho A. (2007). Tolerance and autoimmunity: lessons at the bedside of primary immunodeficiencies. Adv. Immunol. 95, 51–82 10.1016/S0065-2776(07)95002-6 17869610

[B26] Cassani B. Mirolo M. Cattaneo F. Benninghoff U. Hershfield M. Carlucci F. Tabucchi A. Bordignon C. Roncarolo M. G. Aiuti A. (2008). Altered intracellular and extracellular signaling leads to impaired T-cell functions in ADA-SCID patients. Blood 111, 4209–4219 10.1182/blood-2007-05-092429 18218852 PMC2288726

[B27] Chaffee S. Mary A. Stiehm E. R. Girault D. Fischer A. Hershfield M. S. (1992). IgG antibody response to polyethylene glycol-modified adenosine deaminase in patients with adenosine deaminase deficiency. J. Clin. Invest. 89, 1643–1651 10.1172/JCI115761 1569204 PMC443041

[B28] Chan B. Wara D. Bastian J. Hershfield M. S. Bohnsack J. Azen C. G. Parkman R. Weinberg K. Kohn D. B. (2005). Long-term efficacy of enzyme replacement therapy for adenosine deaminase (ADA)-deficient severe combined immunodeficiency (SCID). Clin. Immunol. 117, 133–143 10.1016/j.clim.2005.07.006 16112907

[B29] Churchill P. C. Bidani A. K. (1982). Hypothesis: adenosine mediates hemodynamic changes in renal failure. Med. Hypotheses 8, 275–285 10.1016/0306-9877(82)90124-4 6283327

[B30] Cohen A. Thompson E. (1986). DNA repair in nondividing human lymphocytes: inhibition by deoxyadenosine. Cancer Res. 46, 1585–1588 3485013

[B31] Cunningham-Rundles C. (2011). Autoimmunity in primary immune deficiency: taking lessons from our patients. Clin. Exp. Immunol. 164(Suppl. 2), 6–11 10.1111/j.1365-2249.2011.04388.x 21466546 PMC3087904

[B32] Cuss A. K. Avery D. T. Cannons J. L. Yu L. J. Nichols K. E. Shaw P. J. Tangye S. G. (2006). Expansion of functionally immature transitional B cells is associated with human-immunodeficient states characterized by impaired humoral immunity. J. Immunol. 176, 1506–1516 16424179 10.4049/jimmunol.176.3.1506

[B33] Daikeler T. Tyndall A. (2007). Autoimmunity following haematopoietic stem-cell transplantation. Best Pract. Res. Clin. Haematol. 20, 349–360 10.1016/j.beha.2006.09.008 17448966

[B34] Dave U. P. Akagi K. Tripathi R. Cleveland S. M. Thompson M. A. Yi M. Stephens R. Downing J. R. Jenkins N. A. Copeland N. G. (2009). Murine leukemias with retroviral insertions at Lmo2 are predictive of the leukemias induced in SCID-X1 patients following retroviral gene therapy. PLoS Genet. 5, e1000491 10.1371/journal.pgen.1000491 19461887 PMC2679194

[B35] Davis S. Abuchowski A. Park Y. K. Davis F. F. (1981). Alteration of the circulating life and antigenic properties of bovine adenosine deaminase in mice by attachment of polyethylene glycol. Clin. Exp. Immunol. 46, 649–652 7337981 PMC1536305

[B36] Deaglio S. Dwyer K. M. Gao W. Friedman D. Usheva A. Erat A. Chen J. F. Enjyoji K. Linden J. Oukka M. Kuchroo V. K. Strom T. B. Robson S. C. (2007). Adenosine generation catalyzed by CD39 and CD73 expressed on regulatory T cells mediates immune suppression. J. Exp. Med. 204, 1257–1265 10.1084/jem.20062512 17502665 PMC2118603

[B37] Elliott M. R. Chekeni F. B. Trampont P. C. Lazarowski E. R. Kadl A. Walk S. F. Park D. Woodson R. I. Ostankovich M. Sharma P. Lysiak J. J. Harden T. K. Leitinger N. Ravichandran K. S. (2009). Nucleotides released by apoptotic cells act as a find-me signal to promote phagocytic clearance. Nature 461, 282–286 10.1038/nature08296 19741708 PMC2851546

[B38] Erdmann A. A. Gao Z. G. Jung U. Foley J. Borenstein T. Jacobson K. A. Fowler D. H. (2005). Activation of Th1 and Tc1 cell adenosine A2A receptors directly inhibits IL-2 secretion in vitro and IL-2-driven expansion in vivo. Blood 105, 4707–4714 10.1182/blood-2004-04-1407 15746085 PMC1895001

[B39] Etzioni A. (2003). Immune deficiency and autoimmunity. Autoimmun. Rev. 2, 364–369 10.1016/S1568-9972(03)00052-1 14550878

[B40] Ferrua F. Brigida I. Aiuti A. (2010). Update on gene therapy for adenosine deaminase-deficient severe combined immunodeficiency. Curr. Opin. Allergy Clin. Immunol. 10, 551–556 10.1097/ACI.0b013e32833fea85 20966749

[B41] Fischer A. Le Deist F. Hacein-Bey-Abina S. Andre-Schmutz I. Basile Gde S. De Villartay J. P. Cavazzana-Calvo M. (2005). Severe combined immunodeficiency. A model disease for molecular immunology and therapy. Immunol. Rev. 203, 98–109 10.1111/j.0105-2896.2005.00223.x 15661024

[B42] Fredholm B. B. Dunwiddie T. V. (1988). How does adenosine inhibit transmitter release? Trends Pharmacol. Sci. 9, 130–134 10.1016/0165-6147(88)90194-0 2907698

[B43] Fukunaga A. F. Flacke W. E. Bloor B. C. (1982). Hypotensive effects of adenosine and adenosine triphosphate compared with sodium nitroprusside. Anesth. Analg. 61, 273–278 10.1213/00000539-198203000-00010 7199840

[B44] Gaipl U. S. Sheriff A. Franz S. Munoz L. E. Voll R. E. Kalden J. R. Herrmann M. (2006). Inefficient clearance of dying cells and autoreactivity. Curr. Top. Microbiol. Immunol. 305, 161–176 10.1007/3-540-29714-6_8 16724805

[B45] Gangi-Peterson L. Sorscher D. H. Reynolds J. W. Kepler T. B. Mitchell B. S. (1999). Nucleotide pool imbalance and adenosine deaminase deficiency induce alterations of N-region insertions during V(D)J recombination. J. Clin. Invest. 103, 833–841 10.1172/JCI4320 10079104 PMC408138

[B46] Gaspar H. B. Aiuti A. Porta F. Candotti F. Hershfield M. S. Notarangelo L. D. (2009). How I treat ADA deficiency. Blood 114, 3524–3532 10.1182/blood-2009-06-189209 19638621 PMC2766674

[B47] Gaspar H. B. Cooray S. Gilmour K. C. Parsley K. L. Zhang F. Adams S. Bjorkegren E. Bayford J. Brown L. Davies E. G. Veys P. Fairbanks L. Bordon V. Petropolou T. Kinnon C. Thrasher A. J. (2011). Hematopoietic stem cell gene therapy for adenosine deaminase-deficient severe combined immunodeficiency leads to long-term immunological recovery and metabolic correction. Sci. Transl. Med. 3, 97ra80 10.1126/scitranslmed.3002715 21865538

[B48] Giblett E. R. Anderson J. E. Cohen F. Pollara B. Meuwissen H. J. (1972). Adenosine-deaminase deficiency in two patients with severely impaired cellular immunity. Lancet 2, 1067–1069 10.1016/S0140-6736(72)92345-8 4117384

[B49] Goodnow C. C. (1996). Balancing immunity and tolerance: deleting and tuning lymphocyte repertoires. Proc. Natl. Acad. Sci. U.S.A. 93, 2264–2271 10.1073/pnas.93.6.2264 8637861 PMC39784

[B50] Hacein-Bey-Abina S. Garrigue A. Wang G. P. Soulier J. Lim A. Morillon E. Clappier E. Caccavelli L. Delabesse E. Beldjord K. Asnafi V. Macintyre E. Dal Cortivo L. Radford I. Brousse N. Sigaux F. Moshous D. Hauer J. Borkhardt A. Belohradsky B. H. Wintergerst U. Velez M. C. Leiva L. Sorensen R. Wulffraat N. Blanche S. Bushman F. D. Fischer A. Cavazzana-Calvo M. (2008). Insertional oncogenesis in 4 patients after retrovirus-mediated gene therapy of SCID-X1. J. Clin. Invest. 118, 3132–3142 10.1172/JCI35700 18688285 PMC2496963

[B51] Hasko G. Linden J. Cronstein B. Pacher P. (2008). Adenosine receptors: therapeutic aspects for inflammatory and immune diseases. Nat. Rev. Drug Discov. 7, 759–770 10.1038/nrd2638 18758473 PMC2568887

[B52] Hassan A. Booth C. Brightwell A. Allwood Z. Veys P. Rao K. Honig M. Friedrich W. Gennery A. Slatter M. Bredius R. Finocchi A. Cancrini C. Aiuti A. Porta F. Lanfranchi A. Ridella M. Steward C. Filipovich A. Marsh R. Bordon V. Al-Muhsen S. Al-Mousa H. Alsum Z. Al-Dhekri H. Al Ghonaium A. Speckmann C. Fischer A. Mahlaoui N. Nichols K. E. Grunebaum E. Al Zahrani D. Roifman C. M. Boelens J. Davies E. G. Cavazzana-Calvo M. Notarangelo L. Gaspar H. B. (2012). Outcome of hematopoietic stem cell transplantation for adenosine deaminase deficient severe combined immunodeficiency. Blood. [Epub ahead of print]. 10.1182/blood-2011-12-396879 22791287

[B53] Hauri-Hohl M. M. Keller M. P. Gill J. Hafen K. Pachlatko E. Boulay T. Peter A. Hollander G. A. Krenger W. (2007). Donor T-cell alloreactivity against host thymic epithelium limits T-cell development after bone marrow transplantation. Blood 109, 4080–4088 10.1182/blood-2006-07-034157 17213290 PMC1874583

[B54] Hershfield M. (2005). New insights into adenosine-receptor-mediated immunosuppression and the role of adenosine in causing the immunodeficiency associated with adenosine deaminase deficiency. Eur. J. Immunol. 35, 25–30 10.1002/eji.200425738 15580654

[B55] Hershfield M. S. (1995). PEG-ADA replacement therapy for adenosine deaminase deficiency: an update after 8.5 years. Clin. Immunol. Immunopathol. 76, S228–S232 10.1016/S0090-1229(95)90306-2 7554473

[B56] Hershfield M. S. Buckley R. H. Greenberg M. L. Melton A. L. Schiff R. Hatem C. Kurtzberg J. Markert M. L. Kobayashi R. H. Kobayashi A. L. Abuchowski A. (1987). Treatment of adenosine deaminase deficiency with polyethylene glycol-modified adenosine deaminase. N. Engl. J. Med. 316, 589–596 10.1056/NEJM198703053161005 3807953

[B57] Hershfield M. S. Kredich N. M. Ownby D. R. Ownby H. Buckley R. (1979). In vivo inactivation of erythrocyte *S*-adenosylhomocysteine hydrolase by 2′-deoxyadenosine in adenosine deaminase-deficient patients. J. Clin. Invest. 63, 807–811 10.1172/JCI109367 312296 PMC372019

[B58] Hill N. J. Hultcrantz M. Sarvetnick N. Flodstrom-Tullberg M. (2007). The target tissue in autoimmunity – an influential niche. Eur. J. Immunol. 37, 589–597 10.1002/eji.200636368 17301949

[B59] Hirschhorn R. Candotti F. (2006). “Immunodeficiency due to defects of purine metabolism,” in Primary Immunodeficiency Diseases, eds Ochs H. D. Smith C. I. E. Puck J. M. (Oxford: Oxford University Press), 169–196

[B60] Hirschhorn R. Nicknam M. N. Eng F. Yang D. R. Borkowsky W. (1992). Novel deletion and a new missense mutation (Glu 217 Lys) at the catalytic site in two adenosine deaminase alleles of a patient with neonatal onset adenosine deaminase – severe combined immunodeficiency. J. Immunol. 149, 3107–3112 1401934

[B61] Hirschhorn R. Ratech H. (1980). Isozymes of adenosine deaminase. Isozymes Curr. Top. Biol. Med. Res. 4, 131–157 6115830

[B62] Honig M. Albert M. H. Schulz A. Sparber-Sauer M. Schutz C. Belohradsky B. Gungor T. Rojewski M. T. Bode H. Pannicke U. Lippold D. Schwarz K. Debatin K. M. Hershfield M. S. Friedrich W. (2007). Patients with adenosine deaminase deficiency surviving after hematopoietic stem cell transplantation are at high risk of CNS complications. Blood 109, 3595–3602 10.1182/blood-2006-07-034678 17185467

[B63] Howe S. J. Mansour M. R. Schwarzwaelder K. Bartholomae C. Hubank M. Kempski H. Brugman M. H. Pike-Overzet K. Chatters S. J. de Ridder D. Gilmour K. C. Adams S. Thornhill S. I. Parsley K. L. Staal F. J. Gale R. E. Linch D. C. Bayford J. Brown L. Quaye M. Kinnon C. Ancliff P. Webb D. K. Schmidt M. Von Kalle C. Gaspar H. B. Thrasher A. J. (2008). Insertional mutagenesis combined with acquired somatic mutations causes leukemogenesis following gene therapy of SCID-X1 patients. J. Clin. Invest. 118, 3143–3150 10.1172/JCI35798 18688286 PMC2496964

[B64] Huang S. Apasov S. Koshiba M. Sitkovsky M. (1997). Role of A2a extracellular adenosine receptor-mediated signaling in adenosine-mediated inhibition of T-cell activation and expansion. Blood 90, 1600–1610 9269779

[B65] Isnardi I. Ng Y. S. Srdanovic I. Motaghedi R. Rudchenko S. Von Bernuth H. Zhang S. Y. Puel A. Jouanguy E. Picard C. Garty B. Z. Camcioglu Y. Doffinger R. Kumararatne D. Davies G. Gallin J. I. Haraguchi S. Day N. K. Casanova J. L. Meffre E. (2008). IRAK-4- and MyD88-dependent pathways are essential for the removal of developing autoreactive B cells in humans. Immunity 29, 746–757 10.1016/j.immuni.2008.09.015 19006693 PMC2666307

[B66] Junger W. G. (2011). Immune cell regulation by autocrine purinergic signalling. Nat. Rev. Immunol. 11, 201–212 10.1038/nri2938 21331080 PMC4209705

[B67] Kameoka J. Tanaka T. Nojima Y. Schlossman S. F. Morimoto C. (1993). Direct association of adenosine deaminase with a T cell activation antigen, CD26. Science 261, 466–469 10.1126/science.8101391 8101391

[B68] Klein L. Hinterberger M. Wirnsberger G. Kyewski B. (2009). Antigen presentation in the thymus for positive selection and central tolerance induction. Nat. Rev. Immunol. 9, 833–844 10.1038/nri2669 19935803

[B69] Kohn D. B. (1996). Gene therapy for hematopoietic and immune disorders. Bone Marrow Transplant. 18(Suppl. 3), S55–S58 8971410

[B70] Kohn D. B. (2008). Gene therapy for childhood immunological diseases. Bone Marrow Transplant. 41, 199–205 10.1038/sj.bmt.1705895 17994122

[B71] Koller C. A. Orringer E. P. Berkowitz L. R. Mulhern A. T. (1984). Role of glycolysis in deoxyadenosine induced ATP depletion and dATP accumulation in red cells. Prog. Clin. Biol. Res. 165, 227–239 6334314

[B72] Kono D. H. Haraldsson M. K. Lawson B. R. Pollard K. M. Koh Y. T. Du X. Arnold C. N. Baccala R. Silverman G. J. Beutler B. A. Theofilopoulos A. N. (2009). Endosomal TLR signaling is required for anti-nucleic acid and rheumatoid factor autoantibodies in lupus. Proc. Natl. Acad. Sci. U.S.A. 106, 12061–12066 10.1073/pnas.0905441106 19574451 PMC2715524

[B73] Lainka E. Hershfield M. S. Santisteban I. Bali P. Seibt A. Neubert J. Friedrich W. Niehues T. (2005). polyethylene glycol-conjugated adenosine deaminase (ADA) therapy provides temporary immune reconstitution to a child with delayed-onset ADA deficiency. Clin. Diagn. Lab. Immunol. 12, 861–866 16002636 10.1128/CDLI.12.7.861-866.2005 PMC1182205

[B74] Lappas C. M. Rieger J. M. Linden J. (2005). A2A adenosine receptor induction inhibits IFN-gamma production in murine CD4+ T cells. J. Immunol. 174, 1073–1080 15634932 10.4049/jimmunol.174.2.1073

[B75] Levy Y. Hershfield M. S. Fernandez-Mejia C. Polmar S. H. Scudiery D. Berger M. Sorensen R. U. (1988). Adenosine deaminase deficiency with late onset of recurrent infections: response to treatment with polyethylene glycol-modified adenosine deaminase. J. Pediatr. 113, 312–317 10.1016/S0022-3476(88)80271-3 3260944

[B76] Liao H. Hyman M. C. Baek A. E. Fukase K. Pinsky D. J. (2010). cAMP/CREB-mediated transcriptional regulation of ectonucleoside triphosphate diphosphohydrolase 1 (CD39) expression. J. Biol. Chem. 285, 14791–14805 10.1074/jbc.M110.169599 20178980 PMC2863166

[B77] Luning Prak E. T. (2012). Restoring balance to B cells in ADA deficiency. J. Clin. Invest. 122, 1960–1962 10.1172/JCI63782 22622034 PMC3366416

[B78] Malacarne F. Benicchi T. Notarangelo L. D. Mori L. Parolini S. Caimi L. Hershfield M. Imberti L. (2005). Reduced thymic output, increased spontaneous apoptosis and oligoclonal B cells in polyethylene glycol-adenosine deaminase-treated patients. Eur. J. Immunol. 35, 3376–3386 10.1002/eji.200526248 16276484

[B79] Mandapathil M. Hilldorfer B. Szczepanski M. J. Czystowska M. Szajnik M. Ren J. Lang S. Jackson E. K. Gorelik E. Whiteside T. L. (2010). Generation and accumulation of immunosuppressive adenosine by human CD4+CD25highFOXP3+ regulatory T cells(TREG). J. Biol. Chem. 285, 7176–7186 10.1074/jbc.M109.047423 19858205 PMC2844167

[B80] Marshak-Rothstein A. (2006). Toll-like receptors in systemic autoimmune disease. Nat. Rev. Immunol. 6, 823–835 10.1038/nri1957 17063184 PMC7097510

[B81] Mathis D. Benoist C. (2004). Back to central tolerance. Immunity 20, 509–516 10.1016/S1074-7613(04)00111-6 15142520

[B82] Mazzolari E. de Martiis D. Forino C. Lanfranchi A. Giliani S. Marzollo R. Airo P. Imberti L. Porta F. Notarangelo L. D. (2009). Single-center analysis of long-term outcome after hematopoietic cell transplantation in children with congenital severe T cell immunodeficiency. Immunol. Res. 44, 4–17 10.1007/s12026-008-8022-4 18592143

[B83] Meffre E. (2012). The establishment of early B cell tolerance in humans: lessons from primary immunodeficiency diseases. Ann. N. Y. Acad. Sci. 1246, 1–10 10.1111/j.1749-6632.2011.06347.x 22236425 PMC3925509

[B84] Meffre E. Wardemann H. (2008). B-cell tolerance checkpoints in health and autoimmunity. Curr. Opin. Immunol. 20, 632–638 10.1016/j.coi.2008.09.001 18848883

[B85] Menard L. Saadoun D. Isnardi I. Ng Y. S. Meyers G. Massad C. Price C. Abraham C. Motaghedi R. Buckner J. H. Gregersen P. K. Meffre E. (2011). The PTPN22 allele encoding an R620W variant interferes with the removal of developing autoreactive B cells in humans. J. Clin. Invest. 121, 3635–3644 10.1172/JCI45790 21804190 PMC3163953

[B86] Minguet S. Huber M. Rosenkranz L. Schamel W. W. Reth M. Brummer T. (2005). Adenosine and cAMP are potent inhibitors of the NF-kappa B pathway downstream of immunoreceptors. Eur. J. Immunol. 35, 31–41 10.1002/eji.200425524 15580656

[B87] Montiel-Equihua C. A. Thrasher A. J. Gaspar H. B. (2012). Gene therapy for severe combined immunodeficiency due to adenosine deaminase deficiency. Curr. Gene Ther. 12, 57–65 10.2174/156652312799789253 22348551

[B88] Moratto D. Giliani S. Bonfim C. Mazzolari E. Fischer A. Ochs H. D. Cant A. J. Thrasher A. J. Cowan M. J. Albert M. H. Small T. Pai S. Y. Haddad E. Lisa A. Hambleton S. Slatter M. Cavazzana-Calvo M. Mahlaoui N. Picard C. Torgerson T. R. Burroughs L. Koliski A. Neto J. Z. Porta F. Qasim W. Veys P. Kavanau K. Honig M. Schulz A. Friedrich W. Notarangelo L. D. (2011). Long-term outcome and lineage-specific chimerism in 194 patients with Wiskott–Aldrich syndrome treated by hematopoietic cell transplantation in the period 1980–2009: an international collaborative study. Blood 118, 1675–1684 10.1182/blood-2010-11-319376 21659547 PMC3156052

[B89] Morgan G. Levinsky R. J. Hugh-Jones K. Fairbanks L. D. Morris G. S. Simmonds H. A. (1987). Heterogeneity of biochemical, clinical and immunological parameters in severe combined immunodeficiency due to adenosine deaminase deficiency. Clin. Exp. Immunol. 70, 491–499 3436096 PMC1542189

[B90] Mortellaro A. Jofra Hernandez R. Guerrini M. M. Carlucci F. Tabucchi A. Ponzoni M. Sanvito F. Doglioni C. Di Serio C. Biasco L. Follenzi A. Naldini L. Bordignon C. Roncarolo M. G. Aiuti A. (2006). Ex vivo gene therapy with lentiviral vectors rescues adenosine deaminase (ADA)-deficient mice and corrects their immune and metabolic defects. Blood 108, 2979–2988 10.1182/blood-2006-05-023507 16835374

[B91] Nemazee D. Kouskoff V. Hertz M. Lang J. Melamed D. Pape K. Retter M. (2000). B-cell-receptor-dependent positive and negative selection in immature B cells. Curr. Top. Microbiol. Immunol. 245, 57–71 10.1007/978-3-642-59641-4_3 10533318

[B92] Neven B. Leroy S. Decaluwe H. Le Deist F. Picard C. Moshous D. Mahlaoui N. Debre M. Casanova J. L. Dal Cortivo L. Madec Y. Hacein-Bey-Abina S. De Saint Basile G. De Villartay J. P. Blanche S. Cavazzana-Calvo M. Fischer A. (2009). Long-term outcome after haematopoietic stem cell transplantation of a single-centre cohort of 90 patients with severe combined immunodeficiency: long-term outcome of HSCT in SCID. Blood 113, 4114–4124 10.1182/blood-2008-09-177923 19168787

[B93] Ng Y. S. Wardemann H. Chelnis J. Cunningham-Rundles C. Meffre E. (2004). Bruton’s tyrosine kinase is essential for human B cell tolerance. J. Exp. Med. 200, 927–934 10.1084/jem.20040920 15466623 PMC2213290

[B94] Notarangelo L. D. (2009). Primary immunodeficiencies (PIDs) presenting with cytopenias. Hematology Am. Soc. Hematol. Educ. Program 2009, 139–143 10.1182/asheducation-2009.1.139 20008192

[B95] Notarangelo L. D. Stoppoloni G. Toraldo R. Mazzolari E. Coletta A. Airo P. Bordignon C. Ugazio A. G. (1992). Insulin-dependent diabetes mellitus and severe atopic dermatitis in a child with adenosine deaminase deficiency. Eur. J. Pediatr. 151, 811–814 10.1007/BF01959086 1468454

[B96] Ochs H. D. Buckley R. H. Kobayashi R. H. Kobayashi A. L. Sorensen R. U. Douglas S. D. Hamilton B. L. Hershfield M. S. (1992). Antibody responses to bacteriophage phi X174 in patients with adenosine deaminase deficiency. Blood 80, 1163–1171 1387561

[B97] Ohta A. Madasu M. Kini R. Subramanian M. Goel N. Sitkovsky M. (2009). A2A adenosine receptor may allow expansion of T cells lacking effector functions in extracellular adenosine-rich microenvironments. J. Immunol. 183, 5487–5493 10.4049/jimmunol.0901247 19843934

[B98] Olah M. E. Stiles G. L. (1995). Adenosine receptor subtypes: characterization and therapeutic regulation. Annu. Rev. Pharmacol. Toxicol. 35, 581–606 10.1146/annurev.pa.35.040195.003053 7598508

[B99] Ott M. G. Schmidt M. Schwarzwaelder K. Stein S. Siler U. Koehl U. Glimm H. Kuhlcke K. Schilz A. Kunkel H. Naundorf S. Brinkmann A. Deichmann A. Fischer M. Ball C. Pilz I. Dunbar C. Du Y. Jenkins N. A. Copeland N. G. Luthi U. Hassan M. Thrasher A. J. Hoelzer D. Von Kalle C. Seger R. Grez M. (2006). Correction of X-linked chronic granulomatous disease by gene therapy, augmented by insertional activation of MDS1-EVI1, PRDM16 or SETBP1. Nat. Med. 12, 401–409 10.1038/nm1393 16582916

[B100] Ozsahin H. Arredondo-Vega F. X. Santisteban I. Fuhrer H. Tuchschmid P. Jochum W. Aguzzi A. Lederman H. M. Fleischman A. Winkelstein J. A. Seger R. A. Hershfield M. S. (1997). Adenosine deaminase deficiency in adults. Blood 89, 2849–2855 9108404

[B101] Panther E. Corinti S. Idzko M. Herouy Y. Napp M. La Sala A. Girolomoni G. Norgauer J. (2003). Adenosine affects expression of membrane molecules, cytokine and chemokine release, and the T-cell stimulatory capacity of human dendritic cells. Blood 101, 3985–3990 10.1182/blood-2002-07-2113 12446452

[B102] Parish I. A. Heath W. R. (2008). Too dangerous to ignore: self-tolerance and the control of ignorant autoreactive T cells. Immunol. Cell Biol. 86, 146–152 10.1038/sj.icb.7100161 18227854

[B103] Park J. Y. Shcherbina A. Rosen F. S. Prodeus A. P. Remold-O’Donnell E. (2005). Phenotypic perturbation of B cells in the Wiskott–Aldrich syndrome. Clin. Exp. Immunol. 139, 297–305 10.1111/j.1365-2249.2005.02693.x 15654828 PMC1809280

[B104] Parkman R. Gelfand E. W. Rosen F. S. Sanderson A. Hirschhorn R. (1975). Severe combined immunodeficiency and adenosine deaminase deficiency. N. Engl. J. Med. 292, 714–719 10.1056/NEJM197504032921402 1089883

[B105] Piccirillo C. A. Thornton A. M. (2004). Cornerstone of peripheral tolerance: naturally occurring CD4+CD25+ regulatory T cells. Trends Immunol. 25, 374–380 10.1016/j.it.2004.04.009 15207505

[B106] Poliani P. L. Vermi W. Facchetti F. (2009). Thymus microenvironment in human primary immunodeficiency diseases. Curr. Opin. Allergy Clin. Immunol. 9, 489–495 10.1097/ACI.0b013e3283327e5c 19841578

[B107] Power Coombs M. R. Belderbos M. E. Gallington L. C. Bont L. Levy O. (2011). Adenosine modulates Toll-like receptor function: basic mechanisms and translational opportunities. Expert Rev. Anti. Infect. Ther. 9, 261–269 10.1586/eri.10.158 21342073 PMC4052217

[B108] Ratech H. Hirschhorn R. Greco M. A. (1989). Pathologic findings in adenosine deaminase deficient-severe combined immunodeficiency. II. Thymus, spleen, lymph node, and gastrointestinal tract lymphoid tissue alterations. Am. J. Pathol. 135, 1145–1156 2596574 PMC1880483

[B109] Ratter F. Germer M. Fischbach T. Schulze-Osthoff K. Peter M. E. Droge W. Krammer P. H. Lehmann V. (1996). *S*-adenosylhomocysteine as a physiological modulator of Apo-1-mediated apoptosis. Int. Immunol. 8, 1139–1147 10.1093/intimm/8.7.1139 8757959

[B110] Rogers M. H. Lwin R. Fairbanks L. Gerritsen B. Gaspar H. B. (2001). Cognitive and behavioral abnormalities in adenosine deaminase deficient severe combined immunodeficiency. J. Pediatr. 139, 44–50 10.1067/mpd.2001.115023 11445793

[B111] Saccucci P. Manca Bitti M. L. Bottini N. Rapini N. Piccinini S. D’Annibale F. Chiarelli F. Verrotti A. Bottini E. Gloria-Bottini F. (2009). Type 1 diabetes: evidence of interaction between ACP1 and ADA1 gene polymorphisms. Med. Sci. Monit. 15, CR511–C517 19789510

[B112] Sakaguchi S. (2005). Naturally arising Foxp3-expressing CD25+CD4+ regulatory T cells in immunological tolerance to self and non-self. Nat. Immunol. 6, 345–352 10.1038/nrm1620 15785760

[B113] Sakaguchi S. Yamaguchi T. Nomura T. Ono M. (2008). Regulatory T cells and immune tolerance. Cell 133, 775–787 10.1016/j.cell.2008.05.009 18510923

[B114] Sauer A. V. Aiuti A. (2009). New insights into the pathogenesis of adenosine deaminase-severe combined immunodeficiency and progress in gene therapy. Curr. Opin. Allergy Clin. Immunol. 9, 496–502 10.1097/ACI.0b013e3283327da5 19779332

[B115] Sauer A. V. Brigida I. Carriglio N. Jofra Hernandez R. Scaramuzza S. Clavenna D. Sanvito F. Poliani P. L. Gagliani N. Carlucci F. Tabucchi A. Roncarolo M. G. Traggiai E. Villa A. Aiuti A. (2012a). Alterations in the adenosine metabolism and CD39/CD73 adenosinergic machinery cause loss of Treg cell function and autoimmunity in ADA-deficient SCID. Blood 119, 1428–1439 10.1182/blood-2011-07-366781 22184407 PMC3426348

[B116] Sauer A. V. Morbach H. Brigida I. Ng Y. S. Aiuti A. Meffre E. (2012b). Defective B cell tolerance in adenosine deaminase deficiency is corrected by gene therapy. J. Clin. Invest. 122, 2141–2152 10.1172/JCI61788 22622038 PMC3366410

[B117] Sauer A. V. Mrak E. Jofra Hernandez R. Zacchi E. Cavani F. Casiraghi M. Grunebaum E. Roifman C. M. Cervi M. C. Ambrosi A. Carlucci F. Roncarolo M. G. Villa A. Rubinacci A. Aiuti A. (2009). ADA-deficient SCID is associated with a specific microenvironment and bone phenotype characterized by RANKL/OPG imbalance and osteoblast insufficiency. Blood 114, 3216–3226 10.1182/blood-2009-03-209221 19633200

[B118] Schenk U. Frascoli M. Proietti M. Geffers R. Traggiai E. Buer J. Ricordi C. Westendorf A. M. Grassi F. (2011). ATP inhibits the generation and function of regulatory T cells through the activation of purinergic P2X receptors. Sci. Signal. 4, ra12 10.1126/scisignal.2001270 21364186

[B119] Schenk U. Westendorf A. M. Radaelli E. Casati A. Ferro M. Fumagalli M. Verderio C. Buer J. Scanziani E. Grassi F. (2008). Purinergic control of T cell activation by ATP released through pannexin-1 hemichannels. Sci. Signal. 1, ra6 10.1126/scisignal.1160583 18827222

[B120] Sebastiani G. D. Bottini N. Greco E. Saccucci P. Canu G. Lucarelli P. Gloria-Bottini F. Fontana L. (2006). A study of Adenosine-Deaminase genetic polymorphism in rheumatoid arthritis. Int. J. Immunopathol. Pharmacol. 23, 791–795 20943049 10.1177/039463201002300313

[B121] Serana F. Sottini A. Chiarini M. Zanotti C. Ghidini C. Lanfranchi A. Notarangelo L. D. Caimi L. Imberti L. (2010). The different extent of B and T cell immune reconstitution after hematopoietic stem cell transplantation and enzyme replacement therapies in SCID patients with adenosine deaminase deficiency. J. Immunol. 185, 7713–7722 10.4049/jimmunol.1001770 21057082

[B122] Shou Y. Ma Z. Lu T. Sorrentino B. P. (2006). Unique risk factors for insertional mutagenesis in a mouse model of XSCID gene therapy. Proc. Natl. Acad. Sci. U.S.A. 103, 11730–11735 10.1073/pnas.0603635103 16864781 PMC1518804

[B123] Siaw M. F. Mitchell B. S. Koller C. A. Coleman M. S. Hutton J. J. (1980). ATP depletion as a consequence of adenosine deaminase inhibition in man. Proc. Natl. Acad. Sci. U.S.A. 77, 6157–6161 10.1073/pnas.77.10.6157 6969403 PMC350233

[B124] Simmonds H. A. Goday A. Morris G. S. Fairbanks L. D. Levinsky R. J. (1984). dATP accumulation and ATP depletion in platelets in adenosine deaminase deficiency: significance for the immune response? Biosci. Rep. 4, 809–818 10.1007/BF01138162 6335053

[B125] Simmonds H. A. Levinsky R. J. Perrett D. Webster D. R. (1982). Reciprocal relationship between erythrocyte ATP and deoxy-ATP levels in inherited ADA deficiency. Biochem. Pharmacol. 31, 947–951 10.1016/0006-2952(82)90325-2 7082375

[B126] Sitkovsky M. Lukashev D. Deaglio S. Dwyer K. Robson S. C. Ohta A. (2008). Adenosine A2A receptor antagonists: blockade of adenosinergic effects and T regulatory cells. Br. J. Pharmacol. 153(Suppl. 1), S457–S464 18311159 10.1038/bjp.2008.23 PMC2268051

[B127] Sitkovsky M. V. Lukashev D. Apasov S. Kojima H. Koshiba M. Caldwell C. Ohta A. Thiel M. (2004). Physiological control of immune response and inflammatory tissue damage by hypoxia-inducible factors and adenosine A2A receptors. Annu. Rev. Immunol. 22, 657–682 10.1146/annurev.immunol.22.012703.104731 15032592

[B128] Skalhegg B. S. Landmark B. F. Doskeland S. O. Hansson V. Lea T. Jahnsen T. (1992). Cyclic AMP-dependent protein kinase type I mediates the inhibitory effects of 3′,5′-cyclic adenosine monophosphate on cell replication in human T lymphocytes. J. Biol. Chem. 267, 15707–15714 1379235

[B129] Titman P. Pink E. Skucek E. O’Hanlon K. Cole T. J. Gaspar J. Xu-Bayford J. Jones A. Thrasher A. J. Davies E. G. Veys P. A. Gaspar H. B. (2008). Cognitive and behavioral abnormalities in children after hematopoietic stem cell transplantation for severe congenital immunodeficiencies. Blood 112, 3907–3913 10.1182/blood-2008-04-151332 18645040

[B130] Trobridge G. D. (2011). Genotoxicity of retroviral hematopoietic stem cell gene therapy. Expert Opin. Biol. Ther. 11, 581–593 10.1517/14712598.2011.562496 21375467 PMC3443588

[B131] Utz P. J. Gensler T. J. Anderson P. (2000). Death, autoantigen modifications, and tolerance. Arthritis Res. 2, 101–114 10.1186/ar75 11094420 PMC129993

[B132] Van de Wiele C. J. Joachims M. L. Fesler A. M. Vaughn J. G. Blackburn M. R. Mcgee S. T. Thompson L. F. (2006). Further differentiation of murine double-positive thymocytes is inhibited in adenosine deaminase-deficient murine fetal thymic organ culture. J. Immunol. 176, 5925–5933 16670300 10.4049/jimmunol.176.10.5925 PMC1550651

[B133] Van de Wiele C. J. Vaughn J. G. Blackburn M. R. Ledent C. A. Jacobson M. Jiang H. Thompson L. F. (2002). Adenosine kinase inhibition promotes survival of fetal adenosine deaminase-deficient thymocytes by blocking dATP accumulation. J. Clin. Invest. 110, 395–402 10.1172/JCI200215683 12163459 PMC151094

[B134] Vendetti S. Riccomi A. Sacchi A. Gatta L. Pioli C. De Magistris M. T. (2002). Cyclic adenosine 5′-monophosphate and calcium induce CD152 (CTLA-4) up-regulation in resting CD4+ T lymphocytes. J. Immunol. 169, 6231–6235 12444128 10.4049/jimmunol.169.11.6231

[B135] Vignali D. A. A. Collison L. W. Workman C. J. (2008). How regulatory T cells work. Nat. Rev. Immunol. 8, 523–532 10.1038/nri2343 18566595 PMC2665249

[B136] Waddell D. Ullman B. (1983). Characterization of a cultured human T-cell line with genetically altered ribonucleotide reductase activity. Model for immunodeficiency. J. Biol. Chem. 258, 4226–4231 6339493

[B137] Waldner H. Collins M. Kuchroo V. K. (2004). Activation of antigen-presenting cells by microbial products breaks self tolerance and induces autoimmune disease. J. Clin. Invest. 113, 990–997 10.1172/JCI19388 15057305 PMC379316

[B138] Wardemann H. Nussenzweig M. C. (2007). B-cell self-tolerance in humans. Adv. Immunol. 95, 83–110 10.1016/S0065-2776(07)95003-8 17869611

[B139] Wardemann H. Yurasov S. Schaefer A. Young J. W. Meffre E. Nussenzweig M. C. (2003). Predominant autoantibody production by early human B cell precursors. Science 301, 1374–1377 10.1126/science.1086907 12920303

[B140] Weinberg K. Hershfield M. S. Bastian J. Kohn D. Sender L. Parkman R. Lenarsky C. (1993). T lymphocyte ontogeny in adenosine deaminase-deficient severe combined immune deficiency after treatment with polyethylene glycol-modified adenosine deaminase. J. Clin. Invest. 92, 596–602 10.1172/JCI116626 8349799 PMC294890

[B141] Westerberg L. S. Klein C. Snapper S. B. (2008). Breakdown of T cell tolerance and autoimmunity in primary immunodeficiency – lessons learned from monogenic disorders in mice and men. Curr. Opin. Immunol. 20, 646–654 10.1016/j.coi.2008.10.004 18955138 PMC2605935

[B142] Yip L. Woehrle T. Corriden R. Hirsh M. Chen Y. Inoue Y. Ferrari V. Insel P. A. Junger W. G. (2009). Autocrine regulation of T-cell activation by ATP release and P2×7 receptors. FASEB J. 23, 1685–1693 10.1096/fj.08-126458 19211924 PMC2718802

[B143] Zarek P. E. Huang C. T. Lutz E. R. Kowalski J. Horton M. R. Linden J. Drake C. G. Powell J. D. (2008). A2A receptor signaling promotes peripheral tolerance by inducing T-cell anergy and the generation of adaptive regulatory T cells. Blood 111, 251–259 10.1182/blood-2007-03-081646 17909080 PMC2200810

